# SAVVY Vaginal Gel (C31G) for Prevention of HIV Infection: A Randomized Controlled Trial in Nigeria

**DOI:** 10.1371/journal.pone.0001474

**Published:** 2008-01-23

**Authors:** Paul J. Feldblum, Adesina Adeiga, Rashidi Bakare, Silver Wevill, Anja Lendvay, Fatimah Obadaki, M. Onikepe Olayemi, Lily Wang, Kavita Nanda, Wes Rountree

**Affiliations:** 1 Family Health International, Research Triangle Park, North Carolina, United States; 2 Nigerian Institute of Medical Research, Lagos, Nigeria; 3 College of Medicine, University of Ibadan, Ibadan, Nigeria; Institute of Human Virology, United States of America

## Abstract

**Background:**

The objective of this trial was to determine the effectiveness of 1.0% C31G (SAVVY) in preventing male-to-female vaginal transmission of HIV infection among women at high risk.

**Methodology/Principal Findings:**

This was a Phase 3, double-blind, randomized, placebo-controlled trial. Participants made up to 12 monthly follow-up visits for HIV testing, adverse event reporting, and study product supply. The study was conducted between September 2004 and December 2006 in Lagos and Ibadan, Nigeria, where we enrolled 2153 HIV-negative women at high risk of HIV infection. Participants were randomized 1∶1 to SAVVY or placebo. The effectiveness endpoint was incidence of HIV infection as indicated by detection of HIV antibodies in oral mucosal transudate (rapid test) or blood (ELISA), and confirmed by Western blot or PCR testing. We observed 33 seroconversions (21 in the SAVVY group, 12 in the placebo group). The Kaplan-Meier estimates of the cumulative probability of HIV infection at 12 months were 0.028 in the SAVVY group and 0.015 in the placebo group (2-sided p-value for the log-rank test of treatment effect 0.121). The point estimate of the hazard ratio was 1.7 for SAVVY versus placebo (95% confidence interval 0.9, 3.5). Because of lower-than-expected HIV incidence, we did not observe the required number of HIV infections (66) for adequate power to detect an effect of SAVVY. Follow-up frequencies of adverse events, reproductive tract adverse events, abnormal pelvic examination findings, chlamydial infections and vaginal infections were similar in the study arms. No serious adverse event was attributable to SAVVY use.

**Conclusions/Significance:**

SAVVY did not reduce the incidence of HIV infection. Although the hazard ratio was higher in the SAVVY than the placebo group, we cannot conclude that there was a harmful treatment effect of SAVVY.

**Trial Registration:**

ClinicalTrials.gov NCT00130078

## Introduction

Heterosexual contact accounts for the majority of all human immunodeficiency virus (HIV) infections worldwide, [2] and clear need exists for new technologies to prevent the sexual transmission of HIV. Despite years of effort, an effective HIV-1 vaccine remains elusive. Correct and consistent male condom use has been shown to prevent HIV-1 transmission, [3] but women are often unable to obligate or negotiate the use of condoms by their male partners. Additional strategies to prevent the spread of HIV, particularly for women who are at high risk for HIV acquisition, are crucial. Topical microbicides are products that are designed to inhibit the sexual transmission of HIV and other sexually transmitted infections (STIs). [4] Microbicides could potentially be applied vaginally to prevent both male-to-female and female-to-male transmission; by offering a female-controlled prophylactic option, a microbicide would be an important addition to the prevention toolkit. Unfortunately, no clinical studies to date have demonstrated that these products can prevent HIV infection, [5] and spermicides with the surfactant nonoxynol-9 (N-9) have caused mucosal erosion and ulceration, which may increase the risk of HIV acquisition. [6], [7]

C31G (SAVVY®) has a potent effect on enveloped HIV *in vitro* through a mechanism by which it disrupts the outer membrane. [8] Because its mechanism of action is similar to N-9, [8], [9] some have raised concerns about the safety of C31G. Four Phase 1 studies conducted using three concentrations of C31G (0.5%, 1.0%, 1.7%) assessed signs and symptoms of irritation, epithelial disruption, vaginal colonization, vaginal leakage, and systemic absorption. Signs of irritation were reported, but no serious or severe adverse events related to use of 1.0% C31G gel occurred, and it was less toxic than N-9. [9]–[12] A fifth colposcopy study reported more complaints of discomfort in the C31G users, but similar colposcopic findings of epithelial disruption among users of 1.2% C31G and 2% N-9. [13] The 1.0% concentration was selected by the developer, Biosyn, Inc., for larger-scale testing as SAVVY®. We investigated the safety and effectiveness of 1.0% SAVVY in preventing male-to-female transmission of HIV in a population of young, sexually active Nigerian women at high risk.

## Methods

The protocol for this trial and supporting CONSORT checklist are available as supporting information; see Checklist S1 and Protocol S1.

### Participants

We conducted this randomized, double-blind, placebo-controlled trial between September 2004 and December 2006 in Lagos and Ibadan, Nigeria. We enrolled HIV-antibody negative, non-pregnant women between 18–35 years old who reported more than two coital acts per average week and more than one sex partner in the last three months. Study outreach staff recruited participants from local market areas, bars, hostels, military barracks and colleges, but not brothels. Each study site had two central clinics and 10 peripheral outreach offices spread over various sections of the city. Outreach workers referred interested women to the central clinic.

### Ethics

The study protocol and informed consent forms were approved by: 1) the University of Ibadan/University College Hospital Institutional Review Committee, Ibadan, Nigeria; 2) the Nigerian Institute of Medical Research Institutional Review Board, Lagos, Nigeria; and 3) the Protection of Human Subjects Committee, Family Health International (FHI), NC, USA.

Study counselors read informed consents with each potential participant in the woman's preferred language (English, pidgin or Yoruba). Independent participant advocates witnessed the consent processes for all illiterate women and any other woman who requested one. Prior to signature or fingerprint mark, each woman was asked questions about the information in the consent form to assess her comprehension of the study procedures and purpose.

### Interventions

Consenting women underwent eligibility confirmation and then pregnancy testing; rapid HIV testing with pre- and post-test counseling; physical and pelvic exams; tests for chlamydia, gonorrhea and syphilis; and microscopic examination of vaginal specimens for trichomoniasis, candidiasis, and bacterial vaginosis. Women who were pregnant or HIV rapid test-reactive were screening failures and not enrolled. Women who were confirmed HIV-positive were referred to PEPFAR treatment programs at each institution for monitoring and care. We asked women confirmed eligible at the screening visit to return within 42 days for enrollment. At the enrollment visit, participants received a second informed consent explaining the details of the study. If interested and eligible for the study, participants who signed the enrollment consent form underwent pregnancy testing, rapid HIV testing and family planning counseling and referral to services if requested. Participants were then randomized to receive SAVVY or placebo gel, given a supply of 60 condoms and 60 study gels (to last one month), and scheduled to return for 12 monthly follow-up visits. Staff instructed participants on proper condom use and gel application and told them to return for more condoms and gel if needed. Study staff counseled participants to use condoms at every coital act; that gel effectiveness was unknown; and that they may be receiving a placebo gel that is known not to protect against HIV.

During each monthly follow-up visit at an outreach office, participants answered structured questionnaires including information on recent sexual behavior, condom and gel use, and medical problems or medication use since the previous visit. Participants underwent pregnancy and HIV rapid testing and STI risk-reduction counseling, and received a one-month supply of condoms and study gels. At the 6-month follow-up visit, participants returned to the main study clinic for pelvic exams for STI and saline wet mount examinations of vaginal specimens. We discontinued participants who became pregnant from product use but they remained in the study for monitoring, HIV testing and other data collection, and assessment of pregnancy outcomes. If pregnancy ended during follow-up, a participant could re-start product use. We discontinued participants who were confirmed HIV positive from the study and referred them to the PEPFAR program for care and antiretroviral therapy if needed. If a participant reported any adverse events to the outreach worker during follow-up, she was referred to the study clinic for evaluation and STI testing as needed. If a participant missed a scheduled follow-up visit, study staff made up to 3 attempts to contact that participant and reschedule the visit. After 3 failed contact attempts, no further efforts were made to find her, but her file remained open until study closeout. If the participant did not return to the study before the study was closed, she was considered lost to follow-up at closeout.

### Objectives

The primary objective of this trial was to determine the effectiveness of 1.0% C31G (SAVVY) in preventing male-to-female vaginal transmission of HIV infection among women at high risk.

### Outcomes

The primary measure of effectiveness was infection with HIV, indicated by antibodies in oral mucosal transudate (OMT) (OraQuick® ADVANCE Rapid HIV-1/2 Antibody Test, Orasure Technologies), or by enzyme-linked immunosorbent assay (ELISA) (Genetic Systems™ HIV-1/HIV-2 Plus O ELISA from BioRad) and confirmed by Western blot (Genetic Systems™ HIV-1 Western Blot, BioRad) and/or polymerase chain reaction (PCR) (Roche Amplicor 1.5) from a finger-prick or venous blood specimen.

We tested OMT specimens at screening, enrollment, and then monthly throughout follow-up using OraQuick. If the OraQuick OMT specimen was reactive, we collected blood for ELISA and Western blot and possible PCR testing. At the final follow-up visit all participants received OraQuick OMT and ELISA tests; if either was positive they also had Western blot and PCR tests. We tested stored enrollment specimens using PCR for all participants with HIV infection during follow-up to confirm that seroconversion occurred after enrollment.

We evaluated safety by comparing the incidence of adverse events (AEs), pelvic exam findings and STIs.

### Sample Size

We estimated that a sample size of 2142 participants (1,071 in each treatment group) would yield 80% power to detect a 50% difference in the HIV infection rate (two-sided log-rank test with α = 0.05) between the two groups. With an assumed HIV infection rate in the control group of 5/100 person-years and loss to follow-up of 20%, we anticipated observing 66 HIV infections. [14]–[15] The analysis plan included a blinded assessment of whether additional participants would be needed to observe the required 66 events.

### Randomization—Sequence Generation

We randomized enrolled participants to either SAVVY or placebo using a 1∶1 allocation ratio. An FHI statistician developed the allocation sequence using a computer random number generator and randomly varied permuted-blocks of size 12, 18, and 24, stratified on site.

### Randomization—Allocation Concealment

Six label colors (three SAVVY and three placebo) were used to differentiate the otherwise identically packaged gels. We used sequentially numbered, sealed opaque envelopes to assign participants to one of six color groups after they had qualified for the study and signed the consent form. The randomization envelopes were maintained in a secure office and were not available to study staff until the moment of randomization. Each randomization envelope was used only once.

### Randomization—Implementation

An FHI statistician not otherwise involved with the study developed the allocation sequence. Clinic managers assigned participants to their study groups.

### Blinding

Participants, field study staff, monitors, statisticians, and other FHI staff involved in the trial were not aware of which gel colors were associated with SAVVY or placebo. Both SAVVY and placebo gels were clear, with similar viscosity and pH, dispensed in 3.5 mL doses with identical applicators. The placebo gel was formulated using hydroxyethylcellulose and sorbic acid to minimize any possible effects on study endpoints. It was isotonic and had a pH of 4.4 but with minimal buffering capacity; when mixed with an equal volume of semen, the placebo gel induced a trivial decrease in semen pH (from 7.8 to 7.7). In a phase I clinical trial conducted to assess the safety of the placebo gel, no serious or unexpected AEs were reported following twice-daily intravaginal application for 14 days in healthy sexually abstinent women. [16]

### Statistical Methods

For the primary effectiveness analysis we compared the distribution of HIV-free survival times for the SAVVY and placebo gel groups using a two-sided site-stratified log-rank test. We calculated Kaplan-Meier estimates [17] of HIV infection probabilities by treatment group, pooled across sites. We used a proportional hazards regression model to estimate the hazard ratio and 95% confidence intervals for incident HIV infection, comparing the SAVVY group to the placebo group, with and without controlling for site and other pre-planned baseline variables. We evaluated the homogeneity of the treatment effect across sites, and the proportional hazards assumption, and found no evidence of violation of either at the 0.05 level. We estimated the HIV infection onset date as the midpoint between the date of the first confirmed positive test result and the previous, negative test date. A right censoring time of 380 days was applied. Because the trial was terminated for futility before reaching the number of HIV infections targeted for pre-planned tests of effectiveness, p-values for those analyses should be interpreted with caution. We compared proportions of women with any pelvic exam findings or STIs between treatment groups with a two-sided Mantel-Haenszel Chi-Square Test stratified by site at the 0.05 significance level.

All primary and most secondary analyses were either conducted on the Effectiveness Population (a subset of the Intent-to-Treat (ITT) Population for whom at least one post-enrollment HIV evaluation is available), or the Safety Population which is the subset of the ITT Population who returned after enrollment. The Evaluable Population includes the same participants as the Effectiveness Population but excludes all data collected from a participant after her first documented interruption of product use.

We pre-specified that the independent Data Monitoring Committee (DMC), with access to treatment assignments, would review AEs and primary safety and HIV seroconversion data twice, after approximately 16 and 33 events, respectively. However, testing for early evidence of effectiveness was scheduled to occur only at the second of these two planned looks and again when the target number of events (66) was obtained, controlling for the one planned interim effectiveness analysis. [14]–[15]

## Results

### Recruitment and Participant Flow

**Figure 1:**
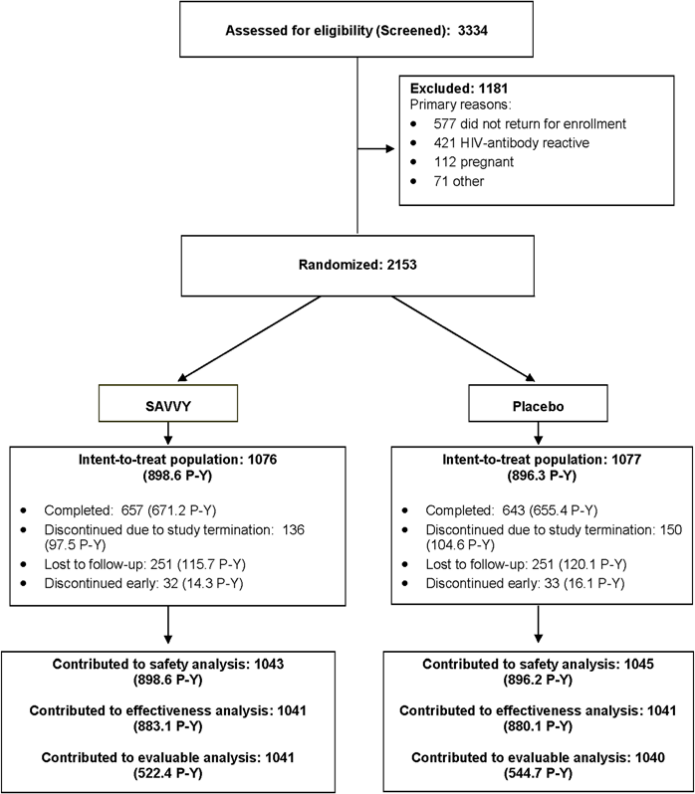
Participant Trial Flow Diagram (P-Y, person years).

The participant screening and enrollment phase began in September 2004 and lasted 19 months. We enrolled 2,153 of the 3,334 screened potential participants (Figure 1). The primary reasons potential participants were not enrolled were because they failed to return for enrollment or were HIV-infected or pregnant at baseline. Follow-up continued until December 2006. Overall loss to follow-up after enrollment was approximately 23% (n = 502), and was the same in each treatment group. Fifty-seven participants never returned for a visit after enrollment, and were excluded from the primary analyses. Due to premature termination of the study (see below), 286 participants completed their final visits before their 12-month visit (Figure 1). Sixty-five additional participants discontinued early, including three participants (one in the SAVVY group and two in the placebo group) who died during follow-up. The three deaths were unrelated to product use. One participant in each treatment group discontinued for a medical reason related to the product.

### Numbers Analyzed

The ITT population comprised 2153 participants who were consented, enrolled and randomized to treatment (1076 in the SAVVY group and 1077 in the placebo group). Effectiveness analyses were conducted on the Effectiveness Population, a subset of the ITT Population with 2082 participants who had HIV testing after enrollment (1041 in both SAVVY and placebo groups; other women never returned for a follow-up visit or had missing data). Safety endpoints were assessed in the Safety Population, the subset of the ITT Population with 2088 participants who returned after enrollment and provided safety data (1043 SAVVY and 1045 placebo). The Evaluable Population included the same participants as the Effectiveness Population (N = 2082) but excluded person-time observed on a participant after her first documented interruption of product use.

### Baseline Data

Most ITT participants in both groups were young (mean age 23.6) and unmarried (Table 1). The most commonly used contraceptive method at baseline was the male condom (SAVVY 72.1%; placebo 68.9%), with oral contraceptives next most frequent (8.9% SAVVY; 8.3% placebo). Some participants also reported dual method use. Most participants had received more than 9 years of education, and were students, or worked in trade or commercial jobs. Other demographic characteristics and medical history were also similar in the two groups (Table 1). About 3.3% of ITT participants were excluded from the Effectiveness Population, and their sociodemographic features (not shown) were virtually the same as the ITT Population.

**Table 1:** Baseline Characteristics (ITT Population).

Characteristic	SAVVY (N = 1076)		Placebo (N = 1077)	
Age		23.5 (3.7)		23.6 (3.8)
Marital status				
Unmarried, not living with a man		923 (85.9)		928 (86.2)
Education				
>9 years		756 (70.3)		777 (72.1)
Occupation				
Student		384 (35.7)		387 (35.9)
Trade/Commerce		384 (35.7)		392 (36.4)
Pregnancy history				
Ever pregnant		800 (74.3)		804 (74.7)
Number of pregnancies		2.2 (1.4)		2.1 (1.4)
Number of vaginal deliveries		0.7 (1.0)		0.6 (1.0)
Contraceptive use				
None		61 (5.7)		71 (6.6)
Condom only		776 (72.1)		742 (68.9)
Dual methods (condom+contraceptive)		90 (8.4)		122 (11.3)
Oral		96 (8.9)		89 (8.3)
IUD		7 (0.7)		7 (0.6)
Injectable		6 (0.6)		10 (0.9)
Other		40 (3.7)		36 (3.3)
Self-reported history of STI		345 (32.1)		321 (29.8)
Previous spermicide use		25 (2.3)		25 (2.3)
Douching		661 (61.4)		653 (60.6)

Data reported as N (%) or mean (SD); SD = standard deviation, IUD = intrauterine device; STI = sexually transmitted infection.

### Sexual Behavior and Adherence

The average number of male partners reported by participants in the last 30 days decreased from 13 (SAVVY group) and 12.5 (placebo group) at the enrollment visit to 9 (SAVVY group) and 8.3 (placebo group) by the 12 month visit. Coital frequency reported by participants in the last week also decreased slightly from 9.6 acts (SAVVY) and 9.5 (placebo) at the enrollment visit to 8.8 acts (SAVVY) and 8.3 acts (placebo) reported at the 12 month visit.

Participants who became pregnant during the trial were asked to stop gel use during pregnancy, but were told they could resume product use after a negative pregnancy test. These participants were included in the intent-to-treat analysis. A total of 272 participants in the SAVVY group and 280 participants in the placebo group became pregnant at least once during the study. The most common reason for product interruption was running out of gel supplies, but pregnancy caused longer interruptions and led to half of all observed person-time off product (not using gel), about 5% of total person-time in both groups.

Participants reported that they used the gel for an average of 78% and 79% of coital acts in the SAVVY and placebo groups, respectively, and reported condoms use for 87% of coital acts in both the SAVVY and placebo groups (Table 2). They reported using both gel and condoms for 69% and 70% of acts in the SAVVY and placebo groups, respectively. Participants reported using neither gel nor condoms for 5% and 4% of acts in the SAVVY and placebo groups, respectively. Gel use reportedly decreased slightly with time in both groups (from 85% at Month 1 to 80% at Month 12 in the SAVVY group, and from 86% at Month-1 to 81% at Month 12 in the Placebo group). Condom use also reportedly decreased over time in both groups (from 92% at Month 1 to 87% at Month 12 in the SAVVY group, and from 91% at Month 1 to 87% at Month 12 for the Placebo group). We calculated that SAVVY or placebo gel was used without condoms for 8–9% of all vaginal sex acts and that condoms were used without gel for 17–18% of all vaginal sex acts. Of the subset of vaginal acts when a condom was not used, gel was reportedly used for 62% of acts (Table 2).

**Table 2:** Estimates of Gel and Condom Use at Follow-Up by Treatment Groups*.

			Mean (median) percentage of reported vaginal sex acts in the last 7 days with						
Treatment	Visit	Mean (Median) number of reported vaginal sex acts	Study gel	Condom	Study gel and a condom	Neither a condom nor study gel	Gel only (without a condom)	Condom only (without study gel)	Study gel when a condom is not used**
SAVVY (N = 1076)	Month 1	10.8 (9.0)	84.7 (100)	91.5 (100)	77.9 (95.1)	2.5 (0.0)	6.8 (0.0)	13.6 (0.0)	66.7 (100)
	Month 6	9.8 (8.0)	75.7 (100)	86.5 (100)	67.2 (85.7)	4.8 (0.0)	8.5 (0.0)	19.3 (0.0)	62.5 (100)
	Month 12	8.8 (7.0)	79.8 (100)	87.3 (100)	70.9 (100)	3.4 (0.0)	9.0 (0.0)	16.5 (0.0)	71.2 (100)
	All FU Visits	10.2 (9.0)	77.9 (100)	87.0 (100)	69.4 (87.5)	4.7 (0.0)	8.5 (0.0)	17.8 (0.0)	62.0 (100)
Placebo (N = 1077)	Month 1	10.0 (8.0)	85.8 (100)	90.5 (100)	78.8 (100)	3.0 (0.0)	6.9 (0.0)	11.9 (0.0)	63.1 (100)
	Month 6	10.0 (8.0)	80.7 (100)	87.0 (100)	71.2 (93.5)	3.7 (0.0)	9.6 (0.0)	15.8 (0.0)	65.3 (100)
	Month 12	8.3 (6.0)	80.5 (100)	86.6 (100)	72.3 (100)	4.0 (0.0)	8.2 (0.0)	14.4 (0.0)	68.9 (100)
	All FU Visits	9.8 (8.0)	79.1 (100)	86.7 (100)	70.3 (90.0)	4.4 (0.0)	8.8 (0.0)	16.6 (0.0)	61.7 (100)

* For each participant and variable of interest (e.g., percentage of vaginal acts where study gel was used in the last 7 days prior to the follow-up visit), we first calculated the participant's mean value of the variable of interest across all of their follow-up visits. (Follow-up visits where women reported a missing number of vaginal sex acts in the last 7 days are excluded from the calculation of a participant's mean value.) The median values of the distributions of these mean values were then obtained for each treatment group.

** **Study gel when a condom is not used** refers to the percentage of condom-free sex acts where gel was used.

### Outcomes and Estimation

Thirteen infections occurred in Lagos, and 20 in Ibadan. The overall HIV incidence rate was 1.87 per 100 person-years (95% confidence interval [CI] 1.29, 2.63). Most seroconversions occurred in younger participants: incidence rates were 2.17 per 100 person-years among women age 18–25 at screening; 1.13 per 100 among women age 26–30; and 0.85 per 100 among women age 31–35.

We observed 33 seroconversions: 21 in the SAVVY group and 12 in the placebo group. The Kaplan-Meier estimates of the cumulative probability of HIV infection at 12 months in the Effectiveness Population were 0.028 in the SAVVY group and 0.015 in the placebo group. The two-sided p-value for the log-rank test of treatment effect was 0.121. The point estimate of the hazard ratio was 1.7 for SAVVY versus placebo (95% CI 0.9, 3.5; Table 3).

**Table 3:** Hazard Ratio of Incident HIV Infection (SAVVY versus Placebo) with and without Adjustment for Baseline Covariates in Effectiveness and Evaluable Populations.

Study Population	Model	Effect	Hazard Ratio (95% CI)	P-value (2-sided)
Effectiveness	M1^a^	SAVVY (vs Placebo)	1.7 (0.9, 3.5)	0.127
	M2^b^	SAVVY (vs Placebo)	1.7 (0.9, 3.5)	0.126
		Ibadan (vs Lagos)	1.1 (0.6, 2.3)	0.708
	M3^c^	SAVVY (vs Placebo)	1.8 (0.9, 3.6)	0.113
		Ibadan (vs Lagos)	0.9 (0.4, 1.9)	0.859
		Ever been pregnant	1.4 (0.6, 3.3)	0.400
		Previous experience using spermicides	2.8 (0.7, 11.9)	0.157
		No. of male partners	0.9 (0.9, 1.0)	0.187
		No. of sex acts not protected by condoms	1.0 (0.7, 1.3)	0.807
		Positive GC or CT at baseline	1.8 (0.4, 7.8)	0.412
Evaluable*	M1^a^	SAVVY (vs Placebo)	1.5 (0.5, 4.8)	0.471
	M2^b^	SAVVY (vs Placebo)	1.5 (0.5, 4.9)	0.459
		Ibadan (vs Lagos)	0.1 (0.0, 1.2)	0.070

a Model M1: treatment effect without adjustment for covariates.

b Model M2: treatment effect adjusted for site.

c Model M3: treatment effect adjusted for site and other baseline covariates, including ever been pregnant, previous experience using spermicides, number of different male partners, number of vaginal sex acts not protected by condoms, and positive GC or CT at baseline.

* Model that adjusted for site and baseline covariates was not implemented for Evaluable population due to small number of events.

### Ancillary Analyses

Adjustment for study site and other pre-planned baseline variables changed the SAVVY hazard ratio little (hazard ratio 1.8; 95% CI 0.9, 3.6; Table 3). None of the selected baseline covariates was significantly associated with HIV infection. Examining pre-planned subgroups of the Effectiveness Population, SAVVY was significantly associated with HIV infection (p = 0.009) in the participants who reported an above median coital frequency during follow-up (median 8–9 in the previous 7 days), and among participants who reported an above median frequency of gel uses (median 6–7 uses in the previous 7 days; p = 0.002). There was suggestive evidence (p = 0.087) that SAVVY was associated with HIV among women who reported above the median number of partners (median 4 partners in previous 30 days). These factors and frequency of condom use were highly correlated.

We repeated the analysis for the Evaluable Population, a subset of the Effectiveness Population, excluding data collected from participants after any documented interruption of product use. Twelve HIV infections occurred in the Evaluable Population (7 in the SAVVY group; 5 in the placebo group). The Kaplan-Meier estimates of the cumulative probability of HIV infection at 12 months in the Evaluable Population were 0.020 in the SAVVY group and 0.013 in the placebo group (2-sided p = 0.449). The unadjusted point estimate of the hazard ratio was 1.5 (95% CI 0.5, 4.8; Table 3).

### Adverse Events

Adverse events (AEs) were reported by 624 of 1043 (60%) participants in the SAVVY group and 636 of 1045 (61%) participants in the placebo group. We found no significant differences in frequency of AEs between treatment groups, either overall or within any specific system organ class. The most frequently reported non-reproductive tract AEs were malaria, abdominal pain, and headache, reported by similar numbers of SAVVY and placebo participants.

Reproductive tract AEs were reported by 327 of 1043 (31%) SAVVY participants and 341 of 1045 (33%) placebo participants, yielding an incidence rate ratio of 0.9 (95% CI 0.8, 1.1). The most frequent self-reported reproductive AEs were vaginal candidiasis, bacterial vaginosis, and vulvovaginitis. In post hoc analyses we evaluated a subgroup of reproductive tract AEs that could be reflective of genital irritation by the study gel, such as vaginal irritation, vaginal burning, self-reported vaginitis, etc. (Table 4). The incidence of these selected AEs was similar in both groups, with an incidence rate ratio of 0.9 (95% CI 0.8, 1.1). We also evaluated these reproductive AEs by categories of self-reported gel use, to investigate a possible relationship between higher use of gel and genital mucosal effects. The incidence of these reproductive system and breast disorders was lower in the SAVVY group (36.8 per 100 person-years) than in the placebo group (42.5 per 100 person-years) among participants with self-reported gel use above the median (data not shown).

**Table 4:** Selected Priority Adverse Events.

	SAVVY (N = 1043)				Placebo (N = 1045)				
System Organ Class/Preferred Term	Number of Events	Number of Women	Percent of Women	IR*	Number of Events	Number of Women	Percent of Women	IR**	Rate Ratio (95% CI) SAVVY vs. Placebo
**Reproductive system and breast disorders** **	**459**	**293**	**28.1**	**38.8**	**444**	**317**	**30.3**	**42.8**	**0.9 (0.8, 1.1)**
....Vaginal candidiasis	168	151	14.5	18.0	152	141	13.5	16.8	1.1 (0.8, 1.4)
....Vaginosis bacterial	112	109	10.5	12.9	117	114	10.9	13.5	1.0 (0.7, 1.3)
....Vulvovaginal pruritus	71	62	5.9	7.1	55	53	5.1	6.1	1.2 (0.8, 1.7)
....Vulvovaginitis trichomonal	39	38	3.6	4.3	47	45	4.3	5.1	0.8 (0.5, 1.3)
....Vaginal discharge	28	27	2.6	3.1	21	20	1.9	2.3	1.3 (0.7, 2.5)
....Genital abscess	7	7	0.7	0.8	11	11	1.1	1.2	0.6 (0.2, 1.8)
....Menstruation irregular	4	4	0.4	0.3	7	7	0.7	0.8	0.4 (0.1, 1.9)
....Vaginal erythema	6	6	0.6	0.7	3	3	0.3	0.3	2.0 (0.4, 12.4)
....Vaginal burning sensation	5	5	0.5	0.6	3	3	0.3	0.3	1.7 (0.3, 10.7)
....Vulvovaginitis	2	2	0.2	0.2	5	5	0.5	0.6	0.4 (0.0, 2.4)
....Genital pain	4	4	0.4	0.4	2	2	0.2	0.2	2.0 (0.3, 22.1)
....Genital rash	4	4	0.4	0.4	2	2	0.2	0.2	2.0 (0.3, 22.1)
....Menorrhagia	3	3	0.3	0.3	3	3	0.3	0.3	1.0 (0.1, 7.4)
....Dyspareunia	3	3	0.3	0.3	2	2	0.2	0.2	1.5 (0.2, 17.9)
....Vaginal haemorrhage	1	1	0.1	0.1	4	4	0.4	0.4	0.2 (0.0, 2.5)
....Menstrual disorder	0	0	0.0	0.0	3	3	0.3	0.3	0.0 (0.0, 2.4)
....Genital lesion	0	0	0.0	0.0	2	2	0.2	0.2	0.0 (0.0, 5.3)
....Vaginal laceration	1	1	0.1	0.1	1	1	0.1	0.1	1.0 (0.0, 78.3)
....Vaginitis	0	0	0.0	0.0	2	2	0.2	0.2	0.0 (0.0, 5.3)
....Oedema genital	1	1	0.1	0.1	0	0	0.0	0.0	-
....Vaginal lesion	0	0	0.0	0.0	1	1	0.1	0.1	0.0 (0.0, 38.9)
....Vaginal ulceration	0	0	0.0	0.0	1	1	0.1	0.1	0.0 (0.0, 38.9)
**Gastrointestinal disorders**	**126**	**109**	**10.5**	**13.0**	**130**	**111**	**10.6**	**13.2**	**1.0 (0.7, 1.3)**
**Pregnancy, puerperium and perinatal conditions**	**0**	**0**	**0.0**	**0.0**	**3**	**3**	**0.3**	**0.3**	**0.0 (0.0, 2.4)**
**Renal and urinary disorders**	**18**	**16**	**1.5**	**1.8**	**14**	**11**	**1.1**	**1.2**	**1.5 (0.6, 3.5)**

* Incidence rate per 100 person-years of follow-up. Excludes events with missing onset dates.

** Selected reproductive system adverse events that could be related to genital irritation. The incidence rate ratio for all adverse events in the reproductive and breast system organ class was 0.9 (0.8, 1.1).

Twenty-eight serious adverse events (SAEs) occurred during the study. Thirteen SAEs were in the SAVVY group and 15 in the placebo group; none was related to study product. Two participants discontinued the study as a result of an SAE; one was hospitalized for appendicitis and another was hospitalized for typhoid enteritis. Information on the three participants who died (one in the SAVVY group and two in the placebo group) was limited. Two participants died at home of unknown causes. Both were noted to be healthy at admission and had not made a study visit for several months prior to death. Another participant had an emergency cesarean delivery at 28 weeks' gestation for pre-eclampsia, and subsequently died of eclampsia.

Participants underwent pelvic examination for chlamydia testing and saline wet mount examinations of vaginal secretions at the 6-month follow-up visit and as needed. No significant differences were seen; of the 1577 participants who had chlamydia tests during follow-up, 4.1% were positive in the SAVVY group and 3.8% in the placebo group. Among the 791 participants who had wet mounts during follow-up, 27.3% of the SAVVY group had bacterial vaginosis compared with 30.4% in the placebo group; 38.0% of the SAVVY group had candidiasis compared with 36.8% of placebo users; and 9.5% of the SAVVY group had trichomoniasis compared with 11.5% of placebo users.

### Study Termination

The DMC met once prior to study initiation. During the trial, the DMC reviewed interim HIV endpoint data (as well as supporting data) for early evidence of SAVVY's effectiveness or signs of potential harm. One interim analysis was planned to look for early evidence of harm when approximately one fourth (i.e., 16) of all expected infections were observed. A second interim analysis was planned to look for convincing evidence of effectiveness when approximately one half (i.e., 33) of all expected infections were observed. Given the low incidence rate, we did the mid-study interim analysis at 29 events, at which time the study statistician estimated that we would need to enroll approximately 1,980 additional participants to identify the 66 HIV infections that would offer the desired study power. Due to concerns about the feasibility of such expansion, we did a conditional power calculation and asked the DMC to review the data and make a recommendation on study continuation. The conditional power analysis showed that the trial was unlikely to provide convincing evidence of effectiveness even with the most optimistic projections. The DMC recommended that the trial be stopped, and we decided to terminate the study prematurely in August 2006.

## Discussion

We prematurely discontinued this study after the third DMC meeting because the HIV incidence among enrolled participants was less than one half the expected rate. Although the hazard ratio was higher in the SAVVY than the placebo group (1.7; 95% CI 0.9 to 3.5), the two-sided p-value for the log-rank test of treatment effect was 0.121 and we cannot conclude that SAVVY was associated with higher levels of HIV infection. Because of our stopping rule for futility and subsequent low power to detect a harmful effect, the absence of a statistically significant finding cannot be taken as evidence of no association. Thus we cannot rule out the possibility that SAVVY increases the risk of HIV among certain users, such as women with above median coital frequency, numbers of partners, or gel use. But those pre-planned subgroup analyses are not conclusive and should be interpreted with caution. [18] The small number of events and the absence of supporting evidence in the similar FHI SAVVY trial in Ghana [19] make this a challenging question that we are unlikely to answer conclusively.

We did not observe significant differences in adverse events among participants receiving SAVVY compared with those receiving placebo, including within the category of reproductive tract/breast disorders. Most of the AEs deemed related to product use were in the reproductive system, and more of them were in the SAVVY group than the placebo group, but they were almost invariably slight or mild. Also, we found no evidence of increased risk of chlamydial or vaginal infections in SAVVY users. Three deaths were reported, but none appeared related to gel use. Our data indicate that use of SAVVY gel does not lead to notable side effects.

During follow-up, participants reported an average of 10 coital acts per week, with an average of 9 sexual partners in the previous 30 days. Reported condom use during follow-up increased from 67% during the last coital act prior to screening to over 90% at the first follow-up visit, remaining over 80% in both groups throughout the follow-up period. Thus, the availability of study gel products did not result in measurably increased risk-taking behaviors among our study participants.

### Interpretation

A number of factors can conspire to render microbicide trials uninformative. Lower than expected HIV incidence, such as we observed in this trial, has been measured in other microbicide trials as well as oral pre-exposure prophylaxis (PrEP) HIV prevention trials. [20] Diminished HIV incidence within a clinical trial may follow from vigorous responses to trial risk-reduction measures, as well as the phenomenon that participants who join clinical trials differ from others in the wider community, [21] in this case a possible inclination to safer behavior. We did not directly measure HIV incidence at the study sites before the trial; even a prospective cohort study would be no guarantee of future incidence since local HIV epidemics are so dynamic.

Second, achieving high adherence with product use is a challenge in HIV prevention studies. More than any other single factor (including low HIV incidence), low adherence compromises the power of a prevention trial. [22] We suspect over-reporting of adherence in this trial: despite SAVVY's purported contraceptive effect, [11] the similar pregnancy rates in the two groups raises doubt that the gels were used as consistently as reported.

A third challenge to measuring microbicide effectiveness is condom use. Although gel was reportedly used during approximately 78% of coital acts in this trial, condom use was even more common and participants were more likely to use gel if a condom was also used. Gel was reportedly used alone (*i.e.,* without a condom) for only about 9% of all coital acts. If true, this too would render it difficult to detect a difference in HIV incidence, since that difference could only arise from a relatively small proportion of potential exposures.

Finally, the occurrence of anal intercourse complicates analysis of topical vaginal agents. Participants were instructed to use the study product only in their vagina. However, at screening 8% of women reported ever having anal sex, and at enrollment approximately 4% of the participants reported having had anal sex and within the last 30 days. HIV transmission studies have indicated that the risk of HIV infection is greater from receptive anal intercourse than receptive vaginal intercourse. [23]–[25] Therefore, some of the seroconversions seen in this vaginal microbicide study could be due to HIV exposure through anal intercourse, with an equal probability of infection regardless of treatment group and resulting bias towards no apparent effect on vaginal transmission.

### Generalizability

These challenges may contribute to the discrepant results reported for recent placebo-controlled microbicide trials. One N-9 trial reported no difference in HIV incidence, [26] while the second reported a higher incidence in the treatment group. [7] The same pattern prevailed in the pair of SAVVY trials, [19] and two as-yet unpublished trials of cellulose sulfate. [Halpern V, Wang L, Obunge O, Ogunsola F, Mehta N, et al. (2007) Effectiveness of cellulose sulfate gel for prevention of HIV: results of the phase III trial in Nigeria. 4th International AIDS Society Conference on HIV Pathogenesis, Treatment, and Prevention. Sydney, Australia, July 22–25, 2007. Abstract WESS302; and Van Damme L, Govinden R, Mirembe F, Guedou F, Solomon S, et al. (2007) Phase III trial of 6% cellulose sulfate (CS) gel for the prevention of HIV transmission. 4th International AIDS Society Conference on HIV Pathogenesis, Treatment, and Prevention. Sydney, Australia, July 22–25, 2007. Abstract WESS301] The wisdom of the U.S. Food and Drug Administration requirement for two trial results significant at the 0.05 level is apparent. [27]

### Overall Evidence

As a new HIV prevention approach, microbicides could be used with other prevention strategies such as condoms to reduce the number of people who become infected with HIV. More powerful Phase 3 studies in higher incidence cohorts are needed to determine conclusively the effectiveness, safety and feasibility of using microbicides for prevention of HIV infection in women. This trial provides no evidence for benefits from SAVVY use.

## Supporting Information

Checklist S1 CONSORT Checklist
(0.06 MB DOC)

Protocol S1 Amended study protocol as implemented in the field
(0.83 MB DOC)
