# Supplementary material for: SAVVY Vaginal Gel (C31G) for Prevention of HIV Infection: A Randomized Controlled Trial in Nigeria
Source: PLoS One. 2008 Jan 23;3(1):e1474. doi: 10.1371/journal.pone.0001474 (PMC2190795; doi:10.1371/journal.pone.0001474)
Supplement: Protocol S1 — Amended study protocol as implemented in the field (0.83 MB DOC) [file pone.0001474.s002.doc]

# **Randomized Controlled Trial of SAVVY and HIV in Nigeria**

Study 9784

**Amendment 5**

**Funded by:** US Agency for International Development

**Sponsor:** BIOSYN, Inc.

1800 Byberry Road

Building 13

Huntington Valley, PA 19006

**Investigational Product:** SAVVY® (C31G)

[Active Ingredients: 1-tetradecamine, N, N-dimethyl-n-oxide N-(carboxymethyl)-, N-dimethyl-1-hexadecamine]

###### IND # 59,283

**Monitor:** Family Health International

PO Box 13590

Research Triangle Park NC 27709

**Project Leader:** Paul Feldblum

Senior Epidemiologist

**FHI Study Clinician:**

Kavita Nanda, MD Associate Medical Director

PO Box 13590

Research Triangle Park NC 27709

Tel: 1.919.544.7040

**Investigators:** Rasheed Ajani Bakare, MBBS, FMCPath, FWACP

Department of Medical Microbiology & Parasitology

College of Medicine

University of Ibadan

Ibadan, Nigeria

Adesina Adeiga, DVM, MSc

Nigerian Institute of Medical Research

Lagos, Nigeria

**Clinical Laboratories**: Sexually Transmitted Infections Laboratory

Department of Medical Microbiology & Parasitology

College of Medicine

University of Ibadan

Ibadan, Nigeria

Molecular Biology and Biotechnology Division

Nigerian Institute of Medical Research

Lagos, Nigeria

# **Randomized Controlled Trial of SAVVY and HIV Nigeria**

Study 9784

**Amendment 5**

I, the Principal Investigator, agree to conduct this study in full accordance with the provisions of this protocol and will comply with all requirements regarding the obligations of clinical investigators as fully outlined in the Declaration of Helsinki and in the Statement of Investigator, which I have also signed. I agree to maintain all study documentation until Family Health International (FHI) advises that it is no longer necessary. I also agree to publish or present data only upon review and after discussion with FHI.

I have read and understand the information in this protocol, including the potential risks and side effects of the product under investigation, and will ensure that all associates, colleagues, and employees assisting in the conduct of the study are informed about the obligations incurred by their contribution to the study

__________________________________ __________________________________

Signature of Principal Investigator Date

Prepared by:

Clinical Research Division

Family Health International

P.O. Box 13950

Research Triangle Park, NC 27709 USA

2224 E. NC Highway 54

Durham, NC 27713 USA

Tel: 1-919-544-7040

FAX: 1-919-544-7261

TABLE OF CONTENTS

[STUDY SUMMARY 6](#__RefHeading___Toc69633701)

[LIST OF ABBREVIATIONS AND ACRONYMS 7](#__RefHeading___Toc69633702)

[1.0 INTRODUCTION 8](#__RefHeading___Toc69633703)

[1.1 Background 8](#__RefHeading___Toc69633704)

[1.2 Rationale 10](#__RefHeading___Toc69633705)

[2.0 STUDY OBJECTIVES 11](#__RefHeading___Toc69633706)

[2.1 Primary Objective 11](#__RefHeading___Toc69633707)

[3.0 TRIAL DESIGN 11](#__RefHeading___Toc69633708)

[3.1 Primary Endpoint 11](#__RefHeading___Toc69633709)

[3.2 Study Design 11](#__RefHeading___Toc69633710)

[3.3 Measures to Minimize Bias 12](#__RefHeading___Toc69633711)

[3.3.1 Randomization 12](#__RefHeading___Toc69633712)

[3.3.2 Allocation concealment 12](#__RefHeading___Toc69633713)

[3.3.3 Masking 12](#__RefHeading___Toc69633714)

[3.3.4 Decoding procedure 12](#__RefHeading___Toc69633715)

[3.4 Study Product 12](#__RefHeading___Toc69633716)

[3.5 Duration of the Study 13](#__RefHeading___Toc69633717)

[3.6 Discontinuation Criteria 13](#__RefHeading___Toc69633718)

[3.6.1 Individual participants 13](#__RefHeading___Toc69633719)

[3.6.2 Study discontinuation 14](#__RefHeading___Toc69633720)

[3.7 Study Product Accountability 14](#__RefHeading___Toc69633721)

[3.8 Randomization Codes 15](#__RefHeading___Toc69633722)

[3.9 Data Directly Recorded on DCFs 15](#__RefHeading___Toc69633723)

[4.0 SELECTION AND WITHDRAWAL OF PARTICIPANTS 15](#__RefHeading___Toc69633724)

[4.1 Eligibility Criteria 15](#__RefHeading___Toc69633725)

[4.2 Loss to Follow-up Procedures 16](#__RefHeading___Toc69633726)

[5.0 STUDY PRODUCTS AND TREATMENTS 16](#__RefHeading___Toc69633727)

[5.1 Products 16](#__RefHeading___Toc69633728)

[5.2 Concomitant Treatments 17](#__RefHeading___Toc69633729)

[5.3 Monitoring of Product Compliance 17](#__RefHeading___Toc69633730)

[6.0 EFFECTIVENESS ASSESSMENT 17](#__RefHeading___Toc69633731)

[6.1 Effectiveness Parameters 17](#__RefHeading___Toc69633732)

[6.2 Methods and Timing of Effectiveness Parameters 18](#__RefHeading___Toc69633733)

[7.0 ADVERSE EVENTS AND REPORTING REQUIREMENTS 20](#__RefHeading___Toc69633734)

[7.1 Adverse Events 20](#__RefHeading___Toc69633735)

[7.2 Relationship of adverse event to study product 21](#__RefHeading___Toc69633736)

[7.3 Reporting adverse events 22](#__RefHeading___Toc69633737)

[7.4 Serious Adverse Events 22](#__RefHeading___Toc69633738)

[7.5 Reporting Serious Adverse Events 23](#__RefHeading___Toc69633739)

[7.6 Social Risk Events 24](#__RefHeading___Toc69633740)

[8.0 STUDY VISIT SUMMARY 24](#__RefHeading___Toc69633741)

[8.1 Screening Visit 24](#__RefHeading___Toc69633742)

[8.2 Enrollment Visit 25](#__RefHeading___Toc69633743)

[8.3 Follow-up Visits 26](#__RefHeading___Toc69633744)

[8.4 Interim Contacts and Unscheduled Visits 27](#__RefHeading___Toc69633745)

[9.0 STATISTICAL SUMMARY 28](#__RefHeading___Toc69633746)

[9.1 Study Size Justification 28](#__RefHeading___Toc69633747)

[9.2 Analysis Plan Summary 28](#__RefHeading___Toc69633748)

[9.2.1 Analysis of effectiveness outcomes 28](#__RefHeading___Toc69633749)

[9.2.2 Analysis of safety outcomes 29](#__RefHeading___Toc69633750)

[9.3 Interim Analysis Plan Summary 29](#__RefHeading___Toc69633751)

[9.3.1 Study size assessment 30](#__RefHeading___Toc69633752)

[9.3.2 Interim analysis of effectiveness outcomes 30](#__RefHeading___Toc69633753)

[10.0 MONITORING PLAN SUMMARIES 31](#__RefHeading___Toc69633754)

[10.1 Clinical Monitoring Plan 31](#__RefHeading___Toc69633755)

[10.2 Data Monitoring Committee 31](#__RefHeading___Toc69633756)

[11.0 DATA MANAGEMENT PLAN SUMMARY 31](#__RefHeading___Toc69633757)

[12.0 PROTOCOL VIOLATIONS 32](#__RefHeading___Toc69633758)

[13.0 STUDY DOCUMENTS 32](#__RefHeading___Toc69633759)

[13.1 Study Initiation 32](#__RefHeading___Toc69633760)

[13.2 Study Conduct 33](#__RefHeading___Toc69633761)

[13.3 Study Completion 34](#__RefHeading___Toc69633762)

[13.4 Site Record Retention and Access to Documents at the Site 34](#__RefHeading___Toc69633763)

[14.0 QUALITY ASSURANCE 34](#__RefHeading___Toc69633764)

[15.0 ETHICS AND RESEARCH INTEGRITY 34](#__RefHeading___Toc69633765)

[15.1 Institutional Review Board Review and Approval 35](#__RefHeading___Toc69633766)

[15.2 Informed Consent 35](#__RefHeading___Toc69633767)

[15.3 Participant Confidentiality 35](#__RefHeading___Toc69633768)

[15.4 Research Integrity 35](#__RefHeading___Toc69633769)

[16.0 PUBLICATION POLICY 36](#__RefHeading___Toc69633770)

[17.0 ADDITIONAL INVESTIGATOR RESPONSIBILITIES 36](#__RefHeading___Toc69633771)

[18.0 REFERENCES 37](#__RefHeading___Toc69633772)

[PROTOCOL ADDENDUM 39](#__RefHeading___Toc69633773)

[APPENDIX 1: STUDY ACTIVITIES CHART 42](#__RefHeading___Toc69633774)

[APPENDIX 2: DIRECTIONS FOR USE OF STUDY GEL 43](#__RefHeading___Toc69633775)

[APPENDIX 3: HANDLING AND STORAGE INSTRUCTIONS 44](#__RefHeading___Toc69633776)

[APPENDIX 4: PARTICIPANT FLOW 45](#__RefHeading___Toc69633777)

# STUDY SUMMARY

# **Randomized Controlled Trial of SAVVY and HIV in Nigeria**

**Study 9784**

**Design:** Phase 3, multi-center, fully-masked, randomized, placebo controlled trial to determine the effectiveness and safety of the 1.0% C31G (SAVVY®) vaginal gel for the prevention of HIV infection.

**Population:** Approximately 2,142 HIV antibody negative participants:

1,071 participants using 1.0% C31G vaginal gel and condoms

1,071 participants using placebo gel and condoms.

**Study Duration:** Approximately 12 months of participant recruitment; 12 months of product use for each participant; approximately 26 total months in the field including screening and close-down.

**Primary Objective:** Determine the effectiveness of 1.0% C31G in preventing male-to-female vaginal transmission of HIV infection among women at high risk.

**Primary Endpoint:** Combined incidence of HIV-1 and HIV-2 infection as determined by detection of HIV antibodies from OMT specimens or by ELISA and confirmed by Western blot and/or PCR.

**Study Sites:**  Lagos and Ibadan, Nigeria

# LIST OF ABBREVIATIONS AND ACRONYMS

AE adverse event

AER adverse event report

API active pharmaceutical ingredient

CT *Chlamydia trachomatis*

DCF data collection form

DMC Data Monitoring Committee

ELISA enzyme-linked immunosorbent assay

FDA US Food and Drug Administration

FHI Family Health International

GC *Neisseria gonorrhoeae*

GCP Good Clinical Practice

HIV human immunodeficiency virus

ICH International Conference on Harmonization

ID identification number

IND Investigational New Drug

IRB Institutional Review Board

KOH potassium hydroxide

N-9 nonoxynol-9

OMT oral mucosal transudate

PHSC Protection of Human Subjects Committee

MIC minimum inhibitory concentration

MTT (3-(4, 5-dimethylthiazolyl-2)-2, 5-diphenyltetrazolium bromide)

RAQA FHI Regulatory Affairs and Quality Assurance Division

RPR rapid plasma reagin

SAE serious adverse event

SAVVY C31G

SEM scanning electron microscope

SOP standard operating procedure

STI sexually transmitted infections

TPHA *Treponema pallidum* Hemagglutination Assay

# 1.0 INTRODUCTION

## 1.1 Background

The Joint United Nations Programme on HIV/AIDS (UNAIDS) estimates that more than 40 million humans are infected with HIV and 5 million newly infected in 2001. AIDS resulting from HIV infection has caused the death of over 20 million people [1]. Heterosexual contact accounts for more than 70% of all HIV-1 infections worldwide [1], and this mode of transmission is increasing in the United States. It is now the predominant route of infection in American women [2].

There is a clear need for new technologies to prevent the sexual transmission of HIV. Despite years of effort, an effective HIV-1 vaccine remains elusive [3]. Correct and consistent male condom use has been shown to prevent HIV-1 transmission [4], but women are often unable to negotiate the use of condoms by their male partners [5-7]. The female condom has recently been marketed as an alternative barrier method [6], but use of this device requires a certain level of skill, and at least the consent of the male partner. Furthermore, the efficacy of the female condom for sexually transmitted infection (STI) prevention is unknown.

Topical microbicides are products that are designed to inhibit the sexual transmission of HIV and other disease pathogens [5-8]. Potentially, they can be applied vaginally to prevent both male-to-female and female-to-male transmission. They also offer a female-controlled prophylactic option in cases in which male condom use cannot be negotiated. Marketed chemical spermicides, which show some activity against STI pathogens in vitro, have been evaluated as topical microbicides. However, no clinical studies have yet demonstrated that these products can prevent HIV infection, and spermicides with N-9 have been shown to cause mucosal erosion and ulceration, which may increase the risk of HIV transmission [9,10].

A vaginal microbicide effective against HIV and other STIs would be an important addition to the current preventive methods. Many of the viral STIs, such as herpes and HIV, have no cure and prevention is the only strategy for controlling the epidemics associated with STIs. Newer products have been formulated, shown promise in pre-clinical studies, and have demonstrated safety profiles in Phase 1 studies that will now permit testing for effectiveness.

C31G (SAVVY®) consists of an equimolar, aqueous mixture of 1-tetradecamine, N, N-dimethyl-n-oxide (myristamine oxide, which is an alkylamine oxide) and N-(carboxymethyl)-, N-dimethyl-1-hexadecamine (cetyl betaine, which is an alkyl betaine), which is buffered to pH 4.8 with citric acid. The two active compounds coalesce into a micelle structure, which is stable and surface active over a wide pH range (4-8).

Several studies have defined SAVVY’s ability to inactivate HIV [11]. As measured by the inhibition of syncitia formation and inhibition of reverse transcriptase assays, a three-log reduction in viral titers was observed after exposure to 0.03% and 0.025% SAVVY respectively. [11]

More recent studies have used HeLa cells, transfected with CD4, co-receptors, and the lac beta galactosidase gene. In these studies, SAVVY was effective at concentrations as low as 0.0125%. Since the sexual transmission of HIV is thought to involve M-tropic strains of HIV-1 rather than T-tropic viruses, the effects of SAVVY on the Bal strain (an M-tropic virus), and strain 89.6 (a dual tropic virus) have been studied. Total inhibition of HIV infectivity was seen at concentrations of 0.0125% for Bal, and 0.00625% for strain 89.6. In total, these studies suggest that SAVVY has a potent effect on enveloped viruses in vitro.

SAVVY is a broad-spectrum antibacterial agent and is active against both gram-positive and gram-negative organisms. SAVVY was also evaluated for antimicrobial activity. [12-13] In a series of experiments to determine the Minimum Inhibitory Concentration (MIC) for a number of indicator microbial and fungal organisms, an optimum pH range of 4.5 to 7 for activity against gram-positive bacteria, and an optimum pH for SAVVY of 5.5 against gram-negative bacteria was demonstrated. At this pH the MIC of SAVVY for *S. aureus* was 0.002%, for *C. albicans* was 0.004%, for *E. coli* was 0.008%, and for *N. gonorrhoeae* was 0.0015%. For comparison, the MICs for N-9 against these organisms were > 1% for *S. aureus*, *C. albicans*, and *E. coli*, and 0.003% for *N. gonorrhoeae*.

Several assays have been used to show SAVVY’s spermicidal activity in comparison with N-9. These assays include computerized Hamilton-Thorn Motility Assay, Sander-Cramer Assay, and use of a fluorescent probe (TO-PRO-1). Studies have shown SAVVY’s ability to reduce total sperm counts, reduce mean progressive velocity and sperm motility; and have measured the rate of cell permeabilization and diffusion into cervical mucus. [14]

SAVVY demonstrated minimal toxicity at 0.001%, as measured by the cell proliferation assay, and no toxicity to mammalian cells at 0.0003%, as measured by the MTT assay. These *in vitro* data are consistent with previous *in vivo* studies (see below), suggesting that SAVVY actually has a subtler mechanism of action.

SAVVY's mechanism of action relates to its absorption by glycolipid cell walls and the subsequent lytic effects on the cell membranes of microorganisms. [15] Molecular modeling has suggested roles for both the polar head and the long chain fatty alkyl portion of the molecule in adsorption to the cell surface and subsequent cell destruction. Scanning electron microscope (SEM) studies have shown that leakage of cell contents occurs rapidly after exposure to SAVVY.

SAVVY functions by a relatively non-specific mechanism of cell membrane lysis. It is unlikely that there will be organisms with any significant degree of resistance to this effect within a microbial population. Thus, the mechanism of generating resistant forms is not expected to be significantly effective with respect to SAVVY. The other possible mechanism, that there are variants which can effectively metabolize/detoxify the antimicrobial agent, is not likely to be very efficient. This is because SAVVY does not have to enter into the microbial cell to have its lytic effect nor pass through any specific metabolic pathway. It only needs to adsorb to the cell wall surface and disrupt the cell membrane. For a microbial cell to metabolize SAVVY prior to its reaching its target, the cell would have to metabolize it via secreted enzymes and these would have to be able to break down fatty alkyl chains. Since microorganisms are not generally known to be able to do this as an extra-cellular function, it seems unlikely that this will be an effective defense mechanism against SAVVY for microbial cells or one that would lead to development of resistance.

Four Phase 1studies have been conducted using three concentrations of SAVVY (0.5%, 1.0%, 1.7%), which included a total of 134 women and 24 men, not including N-9 and nonpoloxamer participants. The first study conducted was a 3-day study, which compared the tolerance and acceptability of various concentrations and formulations of SAVVY. After a formulation was selected a 14-day study was completed, which assessed the effect of test products on irritation of the genitalia (including epithelial disruption as seen on colposcopy), cytological characteristics of vaginal lavage samples, semi-quantitative vaginal cultures and gram stains, vaginal leakage, systemic safety, and acceptability. In addition, plasma and vaginal lavage samples were evaluated for the presence of SAVVY. A post-coital testing study was run in parallel with the 14-day study, which assessed the effectiveness of SAVVY concentrations in preventing sperm from penetrating mid-cycle cervical mucus; genital irritation; acceptability; and SAVVY concentration in cervical mucus. Lastly, a male tolerance study was conducted to ensure that male partners of the women in trials of SAVVY planned for the near future will not be subjected to an undue risk of penile irritation due to exposure to the product and to evaluate whether or not absorption of SAVVY occurred when product was applied to the penis for 7 consecutive days.

All Phase 1 studies showed favorable results and allowed Biosyn, Inc. to select the 1.0% concentration of SAVVY to move into Phase 2 and 3 trials. (See the Investigator’s Brochure for more detail on the studies and outcomes.)

## 1.2 Rationale

Efforts to develop anti-HIV vaccines continue; however, other key means to minimize the spread of HIV must be employed. Male condom use is effective in preventing infection by HIV and other sexually transmitted diseases but social and cultural mores in certain populations may deter this form of transmission control. [16] Therefore, there is a need for additional strategies to prevent the spread of HIV, particularly as it applies to women who are at high risk for HIV acquisition. Topical intravaginal microbicides may offer women a means to control sexual transmission of HIV in settings where they have limited ability to have their male partners use condoms. SAVVY has a good safety profile, will be packaged in individual applicators and is not difficult to use.

Although they are not widely used, spermicides are available in Nigeria. Pharmacies carry a variety of spermicide products in many different formulations including gels, suppositories, and foaming tablets. We will study the safety and effectiveness of SAVVY in a population of young, sexually active women at high-risk for acquiring HIV infection.

# 2.0 STUDY OBJECTIVES

## 2.1 Primary Objective

Determine the effectiveness of 1.0% SAVVY in preventing male-to-female vaginal transmission of HIV infection among women at high risk.

# 3.0 TRIAL DESIGN

This Phase 3 clinical trial will be conducted in compliance with the protocol, FHI SOPs, Good Clinical Practices (ICH/GCP), and the applicable regulatory requirements of the FDA and the site countries.

## 3.1 Primary Endpoint

Combined incidence of HIV-1 and HIV-2 infection as determined by detection of HIV antibodies from OMT specimens or by ELISA and confirmed by Western blot and/or PCR.

## 3.2 Study Design

This is a Phase 3 multi-center, randomized, fully-masked, placebo controlled trial to assess the effectiveness of 1.0% SAVVY in preventing male-to-female vaginal transmission of HIV infection among women at high risk. Eligible participants who are HIV negative, at risk of becoming infected, and are willing to use a vaginal microbicide each time they have intercourse throughout 12 months of study participation will be recruited in Lagos and Ibadan, Nigeria. Approximately 2,142 participants will be enrolled: half of the participants using 1.0% SAVVY with male condoms and the other half using placebo with male condoms. The enrollment phase will last 12 months or until enough participants are enrolled to yield at least 66 new HIV infections.

Enrolled participants will be randomized into either the SAVVY or placebo arm in a 1:1 allocation ratio. Participants will have monthly follow up visits. At each visit they will:

- Receive additional study product
- Undergo a structured interview
- Give urine for a pregnancy test
- Receive HIV risk reduction counseling and
- Give an OMT specimen for HIV testing.

All women will be counseled to use condoms every time they have intercourse to minimize HIV/STI transmission. Participants will also receive contraceptive counseling and referral during screening and enrollment, and may use any additional non-vaginal contraceptive of their choice, although none will be provided as part of this study.

Participants who are HIV-positive at screening or enrollment, or who acquire HIV infection during follow-up, will be counseled and referred to the appropriate medical services.

## 3.3 Measures to Minimize Bias

### 3.3.1 Randomization

A Randomization Manager who is not otherwise involved with the study will develop the allocation sequence using a computer random number generator and randomly varied permuted-blocks. Participants will be randomized to one of six groups (three will be SAVVY and three will be placebo) only after they have qualified for the study and signed the enrollment consent form. Randomization will be stratified by study site and each site will have its own list.

### 3.3.2 Allocation concealment

Group assignments will be concealed in sequentially numbered, sealed opaque envelopes. Trained staff will open the envelopes only after participants are properly enrolled. The randomization envelopes will be maintained in a secure office. They will not be available to the study counselors until the immediate moment of randomization. Each randomization envelope will be used only once.

### 3.3.3 Masking

SAVVY and placebo gels will be identical in packaging, labeling, applicators, and appearance. In order to better mask the study, we will divide the two-arms into three groups each. Therefore, there will be three SAVVY groups and three placebo groups. These groups will be color-coded and participants and staff will not know which groups are SAVVY or placebo. This method works well for logistical purposes and masking purposes.

### 3.3.4 Decoding procedure

The FHI Randomization Manager will keep the randomization code. The Manager will provide the code to the Study Clinician if needed to reveal the masking to the Investigator for emergencies. The Manager will provide the code to the DMC during meetings to review the interim data. The Manager will provide the code to the statisticians when they are prepared to unmask the code after completion of the final masked analysis of the data.

## 3.4 Study Product

C31G (SAVVY®) consists of two different, but chemically related, agents:

1‑Tetradecanamine-N,N‑dimethyl‑N‑oxide and

N‑(Carboxymethyl)‑N,N‑dimethyl-1-hexadecamine

Both of these substances are amphoteric surfactants, but not currently found as API’s together in an approved drug product. They are similar in structure and action to quaternary ammonium compounds. Both ingredients of C31G are used routinely as additives in the cosmetic industry. One component of C31G is a C_14_ alkyl amine oxide (known best as myristamine oxide). The second component is a C_16_ alkyl derivative of N,N-dimethylglycine (cetyl betaine).

SAVVY is a clear vaginal gel and will be administered in a 1.0% 3.5mL dose in a pre-filled, white applicator. The SAVVY and placebo applicators will be overwrapped with a protective white film labeled with “Caution: New Drug – Limited by United States Law to Investigational Use.” SAVVY and placebo will be provided in boxes of 10 prefilled applicators. The outside of the box will be labeled with the Caution statement and have the protocol number indicated.

SAVVY is most effective when administered immediately before intercourse. Participants will be advised to insert SAVVY into their vagina before each and every sex act, but if intercourse does not occur within one hour after application, it should be reapplied. See Appendix 2 for directions of product use and Appendix 3 for storage and handling of SAVVY and the placebo.

The placebo is a clear vaginal gel formulated to match the viscosity and pH of SAVVY and will be administered in a 3.5mL dose via an applicator identical to the applicator used for SAVVY.

## 3.5 Duration of the Study

The screening phase will start 1 month before the enrollment phase and end 1 month before the end of the enrollment phase. Participants will be actively enrolled for approximately 12 months or until enough participants have enrolled to yield 66 new HIV infections. Once enrolled, participants will actively use the study product or placebo for 12 months. Participants will return for monthly follow-up visits. The study will remain open for at least 1 month after the last scheduled follow-up visit to allow follow-up of ongoing AEs, pregnancy outcomes and attempts to find participants that have not returned for their scheduled visits.

## 3.6 Discontinuation Criteria

### 3.6.1 Individual participants

A participant will be discontinued from the study if she has a confirmed HIV seroconversion, although she can continue to visit study offices for counseling and condoms free of charge. In addition, a participant may be discontinued early from the study for any of the following reasons:

- She considers it to be in her best interest or personal reasons
- A physician considers it to be in the participant's best interest because of safety reasons and/or for the well-being of the participant.

Reasons for discontinuation from the study (and study product) will be recorded on the appropriate DCF. **When early discontinuation from the study occurs, every reasonable effort will be made to assess information relevant to** **the endpoints at the time of discontinuation**. This would include attempts to obtain specimens for HIV testing. Withdrawn participants will not be replaced.

**Study product interruption**

Treatment with the study product may be interrupted temporarily or permanently for any of the following reasons:

- The participant ran out of supplies for whatever reason (e.g., she missed a visit); in this case the date of interruption is the date of her last product use
- An adverse event
- The investigator decides to withdraw the study product in the interests of the safety and well being of the participant
- The participant considers it to be in her best interest not to use the study product
- Pregnancy
- Confirmed HIV seroconversion.

If a woman becomes pregnant during the study, she will discontinue use of the study product, but will be encouraged to remain in the study and complete follow-up. She will be tested for HIV each time she completes a follow-up visit. We will collect data on the outcome of the pregnancy as soon as the information is available, even after the end of the study.

Whenever the study product is interrupted, the information will be noted on the appropriate DCF. For all except the first reason listed above, the date of the interruption is the date on which the Investigator decides to interrupt the treatment. Women who interrupt product use due to an adverse event should be followed until the participant has fully recovered. **If possible, participants will be tested for HIV so that the endpoint is determined at the time of product interruption.**

Participants who have had study product interruption for a reason other than confirmed HIV seroconversion may resume study product use and will be given the study product to which they were originally randomized (unless the Investigator determines for safety reasons that the participant should not continue use of the gel). **At the time of product resumption the participant will be tested for HIV so that status of the endpoint is determined**.

### 3.6.2 Study discontinuation

The study will continue until the planned end of the study, or until such time as:

- FHI decides to discontinue the study. FHI may discontinue a site/Investigator due to noncompliance with protocol or regulations
- Biosyn, Inc. decides to suspend or discontinue testing, evaluation, or development of SAVVY
- The IRB decides to discontinue the study.

## 3.7 Study Product Accountability

The study sites will receive a supply of study gels sufficient for the anticipated number of study participants. All supplies must be stored in a limited access area that is securely locked.

The study site will also receive male lubricated (not nonoxynol-9 lubricant) latex condoms to dispense for use with the study gels.

For purposes of inventory accountability, only appropriate site personnel can distribute supplies of the study gels; gels cannot be given to any person not enrolled in this trial.

The study site is required to maintain records of the disposition of each unit of study product received and dispensed, including dates and quantities of distribution to participants, by participant ID number. All unused supplies of the study product must be returned to FHI at the end of the study, or disposed of in a manner specified by FHI.

## 3.8 Randomization Codes

The FHI Randomization Manager will maintain the trial treatment randomization codes. The code for an individual participant will be made available to the FHI Study Clinician, should the need arise to reveal the code. It is anticipated that the unmasking will occur rarely and then only if knowledge of the treatment group would affect health care for the participant. The Investigator will immediately notify the FHI Project Leader if he suspects that unplanned unmasking has occurred.

## 3.9 Data Directly Recorded on DCFs

All DCFs used in the field for this study will have data that is directly recorded on them and will be considered source documents. Other source documents include, but are not be limited to, staff journals, medical notes, screening and enrollment logs, laboratory results, gel dispensing records, consent forms, and reimbursement logs.

# 4.0 SELECTION AND WITHDRAWAL OF PARTICIPANTS

The target population is sexually active women who are at high risk for acquiring HIV. High risk for the purposes of this study includes having intercourse, on average, three times per week and having more than one sexual partner in the last 3 months.

## 4.1 Eligibility Criteria

To be eligible for inclusion in this study, a woman must:

- Be willing and able to give informed consent
- Be at least 18 years old and not more than 35 years old
- Average three coital acts per week
- Have had more than one sexual partner in the last 3 months
- Be willing to use study product as directed
- Be willing to adhere to follow-up schedule
- Be willing to participate in the study for 12 months
- Be willing to report self-medication with antibiotics during study participation
- Be willing to give urine for pregnancy testing, and OMT and blood for HIV testing
- Be willing to not use a spermicide, other vaginal contraceptive, or vaginal lubricant during the study
- Not have a history of adverse reaction to products containing latex
- Not be pregnant or desire a pregnancy during study participation
- Not be an injection drug user
- Not have a gynecological abnormality that may have an impact on the safety and/or response to the study gel
- Not be HIV seropositive.

If a participant has a treatable STI/RTI at screening, she will be treated that day or as soon as possible and she may be enrolled either with untreated infection or while being treated for infection. Eligibility for enrollment is strictly based on HIV infection.

## 4.2 Loss to Follow-up Procedures

If a participant fails to appear for a scheduled follow-up visit, at least three attempts to contact her should be made over the subsequent 30 days. These attempts should be documented in the participant’s study file or other appropriate log. Study staff should make contact attempts for up to three missed visits. After these nine attempts for the three missed visits, no further efforts need be made to find her, but her file should remain open until study closeout.

If the participant does not return to the study before the study is closed, the Final Status Form will be completed at the time of study closeout. The form should indicate that the participant was lost to follow-up. The “lost to follow-up” designation cannot be made for any participant until the closing date of the study.

**Participants will be evaluated for efficacy up until the last follow-up visit.**

# 5.0 STUDY PRODUCTS AND TREATMENTS

## 5.1 Products

**SAVVY**

C31G (SAVVY®) consists of two different, but chemically related, agents:

1‑Tetradecanamine-N,N‑dimethyl‑N‑oxide and

N‑(Carboxymethyl)‑N,N‑dimethyl-1-hexadecamine

Both of these substances are amphoteric surfactants, but not currently found as API’s together in an approved drug product. They are similar in structure and action to quaternary ammonium compounds. Both ingredients of SAVVY are used routinely as additives in the cosmetic industry. One component of SAVVY is a C_14_ alkyl amine oxide (known as myristamine oxide). The second component is a C_16_ alkyl derivative of N,N-dimethylglycine (cetyl betaine).

SAVVY is a clear vaginal gel and will be administered in a 1.0% 3.5mL dose in a pre-filled, white applicator. SAVVY is most effective when administered immediately before intercourse. Participants will be advised to insert SAVVY into their vagina before each and every sex act, but if intercourse does not occur within one hour after application, they should reapply the gel. Participants will be instructed to return for monthly study visits, at which time they will receive a supply of assigned study product. Please see Appendix 2 for directions of product use and Appendix 4 for storage and handling of SAVVY and placebo.

**Placebo**

The placebo gel is formulated to minimize any possible effects — negative or positive — on study endpoints. It is isotonic to avoid epithelial cell swelling or dehydration. It is formulated at a pH of 4.4 but has minimal buffering capacity. When mixed with an equal volume of semen, the placebo gel induced only a trivial decrease in semen pH (from 7.8 to 7.7).

The placebo gel contains hydroxyethylcellulose as a gelling agent, and its viscosity is comparable to that of SAVVY. Hydroxyethylcellulose does not have anti-HIV properties. The gel also contains sorbic acid as a preservative. Sorbic acid has no anti-HIV activity and is readily metabolized by human cells.

Minimal toxicity was observed when the placebo gel was tested in a highly sensitive mouse epithelial toxicity model. When tested in a mouse HSV-2 vaginal challenge model, the placebo afforded no protection when compared to no treatment or when compared to pretreatment with phosphate buffered saline. The placebo also did not enhance susceptibility to HSV-2 when administered 12 hours before vaginal challenge. In contrast, N-9 and other detergent microbicides tested in the same protocol caused a 10-25 fold increase in susceptibility.

## 5.2 Concomitant Treatments

All concomitant medications will be recorded on applicable study DCFs. Medications used for the treatment of AEs that occur during study participation also will be recorded on applicable DCFs. Vaginal medications should not be used concurrently with the study gel. Gel use will be interrupted if a participant needs to use a vaginal medication and the procedures for product interruption will be followed.

## 5.3 Monitoring of Product Compliance

We will assess gel and condom use by interview. We will ask the number of vaginal sex acts the woman had in the 7 days prior to the interview, and among those acts, the number of gels used, the number protected by condoms, the number without condoms or gel, and the number with condoms and gel use together.

# 6.0 EFFECTIVENESS ASSESSMENT

## 6.1 Effectiveness Parameters

The effectiveness of SAVVY against HIV (1 and 2) will be measured by comparing the incidence of HIV antibody detection in the SAVVY group with that in the placebo group.

An incident HIV infection will be defined as follows:

- The presence of HIV antibodies in OMT specimens by OraQuick® rapid test at a follow-up visit, confirmed by Western blot from a dried blood spot specimen, or the presence of HIV antibodies by ELISA and confirmed by Western blot or PCR, and
- The absence of HIV antibodies in OMT specimens by OraQuick rapid test on the sample collected at the previous visit or the presence of an unconfirmed reactive OraQuick result on the sample collected a the previous visit, and
- The absence of a confirmed HIV-positive result for the stored enrollment visit specimen.

## 6.2 Methods and Timing of Effectiveness Parameters

OMT specimens will be tested for HIV-1 and HIV-2 antibodies at screening, enrollment, and then monthly throughout follow-up using OraQuick (OraSure Technologies, Inc.). OraQuick HIV-1 and HIV-2 is a rapid test device that can detect antibodies to HIV within 20 minutes.

If the OraQuick test is reactive, the participant will be told that the test results are preliminary and a blood specimen will be collected for confirmatory ELISA and Western blot testing, and in some cases PCR testing.

Determination of the study endpoint will differ somewhat according to whether the visit is a typical follow-up visit or a final visit. A final visit can refer to either the 12-month visit or an early discontinuation visit.

a) Prior to the 12-month visit, we will do no additional testing of OMT non-reactive participants. Those participants will be asked to return for their next scheduled visit. Those visits do not appear in the table below.

b) Prior to the 12-month visit, an OMT-reactive participant will undergo additional confirmatory testing with ELISA and Western blot, with PCR as needed. That testing is described in the rows of the table below that begin with an OMT+ result in the left column.

c) At the final visit (12-month or early discontinuation), we will perform ELISA on dried blood specimens from all participants. Depending on the combination of OMT and ELISA results, additional testing may be done (see table).

HIV status for the purposes of the statistical analysis, and participant disposition for the purposes of study counseling, are shown in the table on the next page.

| **Combination of HIV Test Results** | | | | **Participant Disposition^1^** | **HIV Status for Analysis** |
| --- | --- | --- | --- | --- | --- |
| OMT - ^2^ | Elisa - | *not done* | *not done* | N/A | HIV - |
| OMT - | Elisa + | WB + | PCR + | N/A | HIV + |
| OMT - | Elisa + | WB - | PCR + | N/A | HIV + |
| OMT - | Elisa + | WB indeterminate | PCR + | N/A | HIV + |
| OMT - | Elisa + | WB indeterminate | PCR - | N/A | HIV - |
| OMT - | Elisa + | WB + | PCR - | N/A | HIV + ^3^ |
| OMT - | Elisa + | WB - | PCR - | N/A | HIV - |
| OMT + | Elisa - | WB - | *not done* | continue | HIV - |
| OMT + | Elisa - | WB + | PCR + | discontinue | HIV + |
| OMT + | Elisa - | WB + | PCR - | discontinue | HIV + ^3^ |
| OMT + | Elisa - | WB indeterminate | PCR + | continue^4^ | HIV + ^5^ |
| OMT + | Elisa - | WB indeterminate | PCR - | continue^4^ | HIV - |
| OMT + | Elisa + | WB - | PCR + | continue^4^ | HIV + |
| OMT + | Elisa + | WB - | PCR - | continue^4^ | HIV - |
| OMT + | Elisa + | WB + | PCR + | discontinue | HIV + |
| OMT + | Elisa + | WB + | PCR - | discontinue | HIV + ^3^ |
| OMT + | Elisa + | WB indeterminate | PCR + | continue^4^ | HIV + |
| OMT + | Elisa + | WB indeterminate | PCR - | continue^4^ | HIV - |

^1^ Disposition prior to the final visit. (First seven rows show 12-month visits, for whom disposition is not applicable.) Women with positive Western blot results should be discontinued from the study and referred to the PEPFAR program for confirmatory testing and management. Women with negative or indeterminate Western blot results should continue and return for the next scheduled follow-up visit and testing.

^2^ OMT– indicates OMT non-reactive; OMT+ indicates OMT reactive.

^3^ Repeat WB and PCR.

^4^ Woman remains in follow-up pending PCR test result.

^5^ OMT+ / PCR+ may not suffice for a positive diagnosis per country-specific guidelines.

The key test result for determining HIV status will be the Western blot result. Positive Western blot results will be termed HIV-positive, regardless of the PCR test result, although we will repeat testing in women who are Western blot positive and PCR negative. Western blot positive participants will be discontinued from the study and gel use, and referred to the local PEPFAR programs for HIV care. PCR testing will not be the sole arbiter of HIV diagnosis. PCR testing will be done even when Western blot results are positive, however, as discrepant results may be of interest for further analysis.

Prior to the final study visit, participant disposition may differ from the ultimate HIV status because the PCR test result is delayed. For example, a participant may be counseled to continue in the study and return for her next follow-up visit, since she does not yet have a positive Western blot result. But if a delayed PCR-positive result is received some weeks later, she may be designated HIV-positive (footnote 4 in table above). If so, the woman will be notified of her HIV-infected status and referred to PEPFAR. The seroconversion date will be the visit at which the PCR-positive specimen was collected.

We will perform PCR using available blood specimens collected at the enrollment visit to verify that participants who became HIV positive during follow-up were truly HIV negative at the time of enrollment. Those participants who are found to have been HIV positive at enrollment will be excluded from the effectiveness analyses.

We recognize that some combinations of positive, negative, indeterminate, or missing results will arise that require individual consideration or additional testing to determine whether a study event has occurred. 1) We will repeat testing for discrepant Western blot and PCR results, as indicated in the table above. 2) Those participants who are likely to be infected but who exhibit hard-to-interpret combinations of results will be referred to the local PEPFAR programs. We will request confirmatory testing by the PEPFAR laboratory in such cases. 3) In our statistical analysis, all such patterns of results will be evaluated in a blind review of the data and without knowledge of gel group. Decisions regarding why each pattern was included or excluded from the set of study events will be documented in the final study report.

# 7.0 ADVERSE EVENTS AND REPORTING REQUIREMENTS

## 7.1 Adverse Events

Study participants will be provided with contact information and instructions to contact the Investigator or designee to report any adverse events (AEs) they may experience, except for life-threatening events, for which they will be instructed to seek immediate emergency care. Based on the results of the Investigator’s assessment, s/he will recommend either continuation or discontinuation of product use. The Investigator also may prescribe or recommend the use of medications or other preparations to treat the AE.

Where feasible and medically appropriate, participants will be encouraged to seek medical care where the site study clinician is based, and to request that the clinician be contacted upon their arrival. With appropriate permission of the participant, records from all non-study medical providers related to AEs will be obtained and required data elements will be recorded on study case report forms. All participants reporting an AE will be followed clinically, until the AE resolves (returns to baseline) or stabilizes.

Study site staff will document on study DCFs all AEs reported by or observed in enrolled study participants regardless of severity and presumed relationship to study product. The Investigators will report annual summary information on all AEs to their IRBs in accordance with US Federal regulations and local IRB requirements.

An AE is any unfavorable and unintended sign, symptom, or disease temporally associated with the use of a medicinal product, whether or not considered related to the medicinal product. Pre-existing events which increase in frequency or severity or change in nature during or because of use of a drug in human clinical trials, will also be considered as adverse events. AEs may also include pre‑ or post‑treatment complications that occur as a result of protocol-mandated procedures (e.g., invasive procedures such as biopsies).

Any medical condition or clinically significant laboratory abnormality with an onset date before the first date of study drug administration is considered to be pre-existing, and should be documented on the DCF noting that it is pre-existing.

Any AE (i.e., a new event or an exacerbation of a pre-existing condition) with an onset date after study product administration up to the last day on study (including the follow-up) should be recorded as an AE on the appropriate DCF.

Site staff must determine the severity of the AE and document it on the appropriate DCF (AE Form). Each adverse event that the participant is aware of should be graded for severity using the following scale:

- **Very slight:** participant was aware of the adverse event on and off.
- **Mild:** participant was aware of the adverse event all of the time, but was still able to do all activities.
- **Moderate:** the participant had to discontinue some activities due to the adverse event.
- **Severe:** the participant was incapacitated by the adverse event and unable to perform normal activities.

An AE **does not** include:

- Medical or surgical procedures (e.g. surgery, endoscopy, tooth extraction, transfusion); the condition that leads to the procedure is an adverse event.
- Pre-existing diseases or conditions present or detected prior to start of study drug administration that do not worsen.
- Situations where an untoward medical occurrence has not occurred (e.g. hospitalization for elective surgery, social and/or convenience admissions).
- Overdose of either study drug or concomitant medication without any signs or symptoms unless the participant is hospitalized for observation.

## 7.2 Relationship of adverse event to study product

The Investigator or designee must determine the relationship of the AE to the product under investigation and document on the appropriate DCF (AE Form). For each AE, an assessment of the relatedness to the test agent should be made using the following scale:

- **Unrelated**: Onset of the AE had no reasonable temporal relationship to administration of the study product or a causal relationship to administration of the study product is biologically implausible or the event is attributed to an alternative etiology.
- **Possibly Related**: Onset of the AE has a reasonable temporal relationship to study product administration and a causal relationship is not biologically implausible.
- **Probably Related**: Onset of the AE has a strong temporal relationship to administration of the study product that cannot be explained by the participant’s clinical state or other factors and a causal relationship is not biologically implausible.
- **Definitely Related**: Onset of the AE shows a distinct temporal relationship to administration of the study product that cannot be explained by the participant’s clinical state or other factors or the AE occurs on rechallenge or the AE is a known reaction to the product or chemical group or can be predicted by the product’s pharmacology.

These criteria in addition to good clinical judgment should be used as a guide for determining the causal assessment. If it is felt that the event is not related to study drug therapy, then an alternative explanation should be provided.

## 7.3 Reporting adverse events

All AEs occurring during the study will be reported on the appropriate DCF (AE Form). The Investigator must provide on this form information on symptoms, time of onset, severity, frequency, product-relatedness, action(s) taken, and participant outcome. FHI’s Study Clinician may request additional information from the site if needed to evaluate the AE.

## 7.4 Serious Adverse Events

A **serious adverse event** (SAE) for a drug or biologic product or medical procedure is defined as any experience that suggests a significant hazard, contraindication, side effect, or precaution. A serious adverse event includes any adverse experience that results in any of the following outcomes:

- Death
- A life-threatening adverse drug experience
- Inpatient hospitalization or prolongation of existing hospitalization
- A persistent or significant disability/incapacity
- A congenital anomaly/birth defect, or
- An important medical event that, based on medical judgment, may jeopardize the patient or subject and may require intervention to prevent one of the outcomes listed above.

Examples of such events are:

- Intensive treatment in an emergency room or at home for allergic bronchospasm
- Blood dyscrasias or convulsions that do not result in hospitalization
- Development of drug dependency or drug abuse.

**Clarification of SAEs:**

- Death is an outcome of an adverse event, and not an adverse event in itself.
- All deaths, regardless of cause, must be reported for participants on study and for deaths occurring within 30 days of last study product use or within 30 days of last study evaluation, whichever is longer.
- “Occurring at any dose” does not imply that the participant is using study product at the time of the event. Product use may have been interrupted temporarily prior to the onset of the SAE, but may have contributed to the event.
- “Life-threatening” means that the participant was at immediate risk of death from the event as it occurred. This does not include an event that might have led to death, if it had occurred with greater severity.
- Complications that occur during hospitalizations are AEs. If a complication prolongs hospitalization, it is a SAE.
- “Inpatient hospitalization” means the participant has been formally admitted to a hospital for medical reasons, for any length of time. This may or may not be overnight. It does not include presentation and care within an emergency department unless the participant is “admitted”.
- The Investigator should attempt to establish a diagnosis of the event based on signs, symptoms and/or other clinical information. In such cases, the diagnosis should be documented as the AE and/or SAE and not the individual signs/symptoms.

## 7.5 Reporting Serious Adverse Events

All serious adverse events must be reported to FHI ***within 24 hours*** of the study site becoming aware of the problem. The Investigator should complete a FHI SAE Report Form and fax it to:

***FHI Regulatory Affairs and Quality Assurance***

Phone: (919) 544-7040, ext. 351

FAX: (919) 544-1308

Email: SAE@fhi.org

In cases in which a SAE Report Form cannot be faxed within 24 hours, the Investigator may report the SAE via telephone or e-mail; however, a SAE Report Form must be completed as soon as possible after the informal report. FHI Regulatory Affairs and Quality Assurance (RAQA) staff will forward the SAE to the Study Clinician and the Project Leader for processing. The Study Clinician will make sure to update this information as soon as he/she receives a complete SAE Report Form from the Investigator.

The FHI Study Clinician and FHI Chief Medical Officer will complete the FHI SAE Review Form within 5 days of receipt of the SAE Report Form (including interim Report Form) from the site.

If the SAE is unexpected, and related to the use of an investigational product, the FHI Project Leader/Study Clinician will alert Biosyn immediately of receipt of the SAE Report Form that an SAE has occurred that may be reportable to the regulatory agency. The FHI Project Leader/Study Clinician will process and report all SAEs to Biosyn and PHSC in accordance with the SOP#301 and/or conditions specified in the Memorandum of Understanding between FHI and Biosyn.

The Investigator will be responsible for immediate reporting of all SAEs to FHI RAQA staff as specified above, and prompt reporting of all SAEs to the local IRB.

## 7.6 Social Risk Events

No social risk events are expected from participation in this study. However, if the Investigator learns of a social risk event, he will report the event to FHI RAQA on the Social Risk Event Form.

# 8.0 STUDY VISIT SUMMARY

Study staff will be stationed throughout the city to identify potential study participants. When the staff has identified a potential participant, she will give a brief description of the study, conduct preliminary screening for eligibility, and ascertain her interest. Staff will refer interested eligible women to the study clinic.

Participants will make visits to a study clinic at screening, enrollment, and at or after 6-months. In addition, they will make 12 monthly follow-up visits. See Appendix 1 for a table of study procedures and timing of activities and Appendix 4 for participant flow through the study.

## 8.1 Screening Visit

The study, including potential risks and benefits of participation, will be explained to prospective participants during screening. This will be a general overview of the study purpose and procedures. Each woman will be asked to sign (or provide other mark) a consent form for the screening procedures, and will be given a copy of the consent form to take home. If the participant cannot read, a witness will be present at the time of the study explanation and the consent process, and will witness the signature or mark of the participant.

All volunteers will be assigned an ID number (for the purpose of tracking their lab tests), and evaluated to determine their eligibility according to the participation eligibility criteria.

The following will be done:

- An interview with the woman to make sure she understands the study schedule and is willing to comply with study requirements
- Consent process
- Pre-test HIV counseling
- Contraceptive counseling
- A urine pregnancy test
- Blood draw for syphilis testing (as part of the standard of care for HIV prevention)
- OMT specimen taken for HIV testing; a dried blood spot specimen will be taken for the confirmation tests if the OraQuick OMT test is reactive
- Post-test HIV counseling
- Pelvic examination
- GC and CT testing

If a woman is HIV positive, she may return to the clinic and speak with a study counselor for the duration of the study. The study staff will refer her to local resources for HIV positive persons to receive additional support and care.

The women will be asked to return in 4 weeks (± 15 days) for the enrollment visit. If they are HIV negative and meet the other enrollment criteria, they may be enrolled at that time. Any curable STI diagnosed will be treated. Whenever possible, single dose directly observable treatments will be used for all treatments in this study. The STI treatments approved by the local Drug Board and Ministry of Health will be used.

All women will be instructed in proper condom use and asked to use them until the enrollment visit.

***Pelvic exam***

The following examination and specimen collection sequence will be followed:

- Inguinal lymph nodes palpated and enlargement and tenderness noted
- Vulva and perineum visually inspected for breaks in epithelial integrity, warts, or other lesions.
- Vaginal introitus inspected for discharge and odor
- A speculum lubricated with water will be inserted into the vagina
- Inspection of the vagina and cervix
- Vaginal pH measured by holding a pH strip against the lateral vaginal wall with a cotton tipped applicator and compared to the pH chart provided with the strips
- A vaginal swab from the posterior fornix placed in a test tube with a few drops of normal saline for preparation of the wet-mount (saline and potassium hydroxide)
- An endocervical swab for the GC and CT testing
- A bimanual pelvic examination performed

## 8.2 Enrollment Visit

If the woman meets all eligibility criteria, she will receive a detailed explanation of study procedures, and counseled in the importance of returning for follow-up. She will be asked to sign an enrollment consent form, and will be given a copy to take home. She will then be enrolled in the study and randomly assigned to one of the study groups.

***Contact information***

For each participant, clinic staff will obtain contact information. The study site will develop its own Locator Form, and determine the best way to collect this information for its own study population. In the event that a participant misses a scheduled appointment, the clinic staff will try to establish communication with the participant through all possible means (e.g., telephoning if this is possible, writing to the participant and contacts, and/or visiting the participant’s home or workplace). These attempts will be documented in the participant’s file or appropriate log. The need to return for all scheduled follow-up visits will be emphasized to all study participants at every visit.

***Counseling***

The counselor will review study product use, and methods of HIV/STI transmission and prevention, including abstinence and monogamy messages. Contraceptive counseling will be done. The use of condoms with all partners, including primary sexual contacts, will be emphasized to all participants. Staff will also stress that this is a controlled trial of a product of unknown effectiveness in the prevention of HIV transmission and that condoms should be used for all sexual contacts with all partners. This information will be repeated at each follow-up counseling session for both groups.

***Laboratory testing***

Staff will collect an OMT specimen for HIV testing and the results of the OraQuick HIV test will be known before randomization takes place. All women will give blood specimens for storage and possible confirmatory HIV testing. A urine pregnancy test will be conducted and pregnant women will be excluded from study participation.

***Study product***

Each participant will receive a supply of SAVVY or placebo along with condoms. Participants will receive supplies in quantities anticipated to last until the next visit. A participant will be instructed to return between scheduled visits to obtain more supplies if needed. A product-dispensing log will be maintained by the clinic and field staff to track all gel products.

***Pelvic* *examination***

A pelvic examination will only be done if the participant has symptoms or a problem that needs to be checked. If a wet mount is positive for *T. vaginalis,* clinical signs or symptoms of a yeast infection with yeast seen on a KOH wet mount, or bacterial vaginosis (Amsel criteria), the woman can be enrolled while starting treatment. Treatment will be recorded on the appropriate DCF and treatment log.

***HIV confirmation at screening and enrollment***

If the OraQuick test is reactive during screening or enrollment, the participant will be told that the test results are preliminary, and a blood specimen will be collected by venipuncture for ELISA and Western blot testing. If one or both of the ELISA and Western blot tests is positive, the woman will be designated HIV positive. Regardless of her final HIV status, however, a woman with a reactive OraQuick result at screening or enrollment will be excluded from trial participation. Women designated HIV-positive will be referred to treatment programs.

## 8.3 Follow-up Visits

Monthly follow-up visits are scheduled throughout the study follow-up period on the same date as the participant’s enrollment date (i.e., on the monthly “anniversary” of the enrollment date), and not the date of her last visit. Acknowledging that it will not always be possible to complete follow-up visits on the targeted dates, visits may be completed within a 30-day window around the target date (i.e., ± 15 days from the target date). For participants who do not complete scheduled visits within the allowable window, the visit will be considered “missed” and the appropriate DCF will be completed to document the missed visit.

Each participant will have a follow-up visit at an outreach office site convenient for her. Each outreach worker will be assigned to a site and will use the site as a base of operation. The outreach worker is responsible for monthly counseling, interviewing, re-supplying the participants with products, testing for HIV and pregnancy, and, if necessary, home visits to ensure that each participant completes the study. Activities at the monthly visit include:

- A questionnaire will be administered to collect information on use of gel and condoms, and any medical symptoms
- Participants will be counseled about gel and condom use
- Participants will be re-supplied with gel and condoms in an amount sufficient to last until the next follow-up visit
- Participants will provide an OMT specimen for HIV testing
- Participants will give a urine sample for pregnancy testing

If at any visit, the staff person or the study participant thinks that a pelvic examination is necessary or that an AE has occurred, study staff will refer the participant to a study clinic for an examination by a study nurse or a study physician. Also, each participant will be scheduled for a clinic visit at 6 months to perform STI and microbiology testing, and to receive a pelvic examination. If confirmatory HIV testing is needed, the participant will be referred to the clinic for blood collection.

Finally, the participant’s final or discontinuation visit will preferably be done at the clinic. An acceptability questionnaire will be administered and a blood specimen will be collected for HIV status confirmation. Participants will be asked to return to the clinic after their final visit for HIV test results. In some cases, a participant may be asked to return to the clinic for additional testing after 12 months if the HIV results remain unclear.

**STI**

Whenever a curable STI is diagnosed, treatment will be given to the participant and recorded on the DCF. If one of the laboratory tests is positive without clinical evidence of an STI and/or if a prescribed treatment has to be adjusted after laboratory results become available, the participant will be contacted and treated if necessary (study product can be continued). Where possible, the participant’s partners will be referred for treatment.

## 8.4 Interim Contacts and Unscheduled Visits

The participant or the Investigator may request an interim contact or an unscheduled visit at any time during the study. All interim contacts and unscheduled visits will be documented in the participants’ study records and on applicable DCFs.

When an interim contact or unscheduled visit occurs in response to AEs experienced by study participants, study staff will assess the reported event clinically and provide or refer the participant to appropriate medical care. All AEs associated with genital symptoms will be evaluated according to the pelvic exam procedures described for follow-up visits and other procedures as deemed necessary by the Investigator, and diagnosis and follow-up of any observed abnormalities will proceed according to the procedures described there and in the “Adverse Events” section.

Interim visits are distinguished from adverse event visits in that no health-related reaction, effect, or abnormality is reported. Some examples of reasons for an interim visit are:

- To get more product or condoms
- To ask questions of study staff
- To discuss problems with study compliance
- To report the outcome of a pregnancy conceived during the study
- Interim examination – e.g., an examination requested with no complaints or symptoms.

# 9.0 STATISTICAL SUMMARY

## 9.1 Study Size Justification

A two-sided log-rank test with an overall sample size of 2,142 participants (of which 1,071 are in the SAVVY group and 1,071 are in the placebo group) achieves at least 80% power at a 0.05 significance level to detect a 50% reduction in the HIV infection rate if the rate of infection in the control group is 5/100 person-years and loss to follow-up is at most 20% in both groups. These calculations were conducted using the PASS© 2000 software, which assumes exponential models for the times to HIV detection and losses to follow-up. Approximately 66 total HIV infections are required to achieve the desired power.

## 9.2 Analysis Plan Summary

A detailed analysis plan that covers both the final analysis and the planned interim analysis will be developed and approved prior to the initiation of the study. The following is a summary of the planned analyses. Any deviations to be made from this summary plan will be documented in the detailed analysis plan.

All primary and most secondary analyses will be conducted on an intent-to-treat basis. However, in order to assess the effectiveness and safety of SAVVY among those participants who complied with key aspects of the study protocol and to evaluate the potential impact of treatment on loss to follow-up, various additional analysis populations may be defined in the detailed analysis plan prior to study initiation. Any key decisions regarding the timing of outcomes, the appropriateness of test statistics or model assumptions, or any other statistical issues will be made in a masked review of the data (i.e., masked to the true randomization groups). Unmasking with respect to the true randomization groups will only be done for the final interpretation of the results, unless otherwise required by the data monitoring committee (DMC) (see section 10.2).

### 9.2.1 Analysis of effectiveness outcomes

The effect of SAVVY on the time to HIV detection will be determined using two one-sided log-rank tests stratified by site. The type one error rate for the one-sided test with alternative hypothesis that SAVVY protects against HIV acquisition will be carried out at the 0.025 level and adjusted to account for the interim analysis described below.[[1]](#footnote-2) Secondarily, proportional hazards regression models will be used to estimate the hazard rate ratio, along with a 95% confidence interval, comparing the SAVVY group to the placebo group for the HIV outcome, controlling for site and any important baseline prognostic variables. A list of the baseline prognostic variables to be included in the models will be provided in the detailed analysis plan prior to study initiation. The homogeneity of the treatment effect across sites will be investigated by introducing a treatment-by-site interaction term to the model. Crude incidence rates, rate ratios, and 95% confidence intervals will be calculated and will be based on the ratio of the number of new infections to the total length of treatment exposure in the interval. Time to HIV detection, in days, will be computed as the difference between the date of HIV detection, estimated using the midpoint between the dates of the first positive HIV test and the preceding negative HIV test, and the enrollment date, plus one.

In addition to the primary analysis, Investigators have an interest in comparing pregnancy rates between the SAVVY and placebo groups. Crude pregnancy rates and corresponding 95% confidence intervals will be calculated by randomization group, and stratified by contraceptive method used at baseline. Outcomes of all pregnancies that occur will be ascertained on worksheets designed for that purpose (see Protocol Addendum, p.37). No hypothesis tests regarding pregnancies are planned. Because this is not a study of the contraceptive effectiveness of SAVVY and because participants will not be discontinued after they develop a desire to become pregnant during the study, any results regarding this outcome will be interpreted with extreme care.

### 9.2.2 Analysis of safety outcomes

On a quarterly basis throughout the trial, adverse events will be coded using the MedDRA system in a collaborative effort by the study clinician and data management staff. The AEs will be summarized by body system and pooled over treatment arms (i.e., completely blinded). Prevalence and incidence rates, pooled over treatment arms, will be calculated for certain important or interesting AEs or groups of AEs (to be determined in collaboration with the project leader and study clinician). These summaries will be used for monitoring purposes only.

## At the end of the trial, AEs occurring during the study will be summarized in frequency tables (including both the number of each type of AE and the number of distinct participants with each type of AE), by body system and by randomization group.

## 9.3 Interim Analysis Plan Summary

Two interim analyses for the use of the DMC are planned to evaluate the safety and effectiveness of SAVVY relative to the placebo in terms of preventing HIV infection. The first interim analysis will take place after approximately one quarter of anticipated HIV infections (i.e., 16 infections) have occurred and will focus exclusively on safety (with HIV as the primary safety endpoint), and the second interim analysis will take place after approximately one half of anticipated HIV infections (i.e., 33 infections) have occurred and will evaluate both the safety and effectiveness of SAVVY. All analyses will be conducted on a masked basis as follows. Project biostatisticians will prepare and verify the interim analysis programs using randomly generated dummy treatment assignments that have no relationship to the true treatment assignments. An independent biostatistician not otherwise involved with the study will subsequently incorporate the masked treatment codes (i.e. “Drug A” or “Drug B”) into the analysis programs for reporting of interim results. At the request of the DMC, the unmasked treatment codes will be provided separately by an FHI statistician (the Randomization Manager) who is not otherwise involved in the study. The interim analyses will include descriptive statistics (e.g., frequency distributions for categorical variables and means, standard deviations, and ranges for continuous variables) of baseline variables, compliance with intervention, discontinuation rates, intervention-associated morbidity (e.g., epithelial disruptions), and HIV incidence. It is recognized that scheduling restrictions of the study monitors and the DMC may preclude performing the interim analyses at precisely the study quarter- and halfway points, and methods will be employed that accommodate these restrictions.

### 9.3.1 Study size assessment

Prior to the second interim analysis (i.e., after approximately 33 events) of the HIV outcome and prior to examining event rates by masked randomization group, the lead statistician will make recommendations (based on data pooled across randomization groups) regarding adjustment of study size in accordance with the requirement of observing approximately 66 new HIV infections by the conclusion of the study. If either the pooled HIV infection rate or the follow-up rate is substantially lower than anticipated when the study was designed (0.0375 and 0.80, respectively), then consideration will be given to increasing the total study size. Under no circumstances will the statistician recommend decreasing the overall study size. The observed treatment effect at the time of the interim analysis will not be considered as part of this evaluation. Hence no type I error rate adjustment is required to account for this study size assessment.

### 9.3.2 Interim analysis of effectiveness outcomes

Considerations for early termination of the study will be made primarily based on comparisons of the HIV event rates between treatment groups. At the first interim analysis after approximately 16 events, we will ask that the DMC only consider stopping the study for evidence of a potentially harmful SAVVY effect (i.e., evidence that SAVVY enhances HIV transmission), as determined by a one-sided log-rank test at the 0.10 significance level. At the second interim analysis after approximately 33 events, we will ask that the DMC allow consideration for stopping the study either due to evidence of a potentially harmful SAVVY effect, as determined by a one-sided log-rank test at the 0.10 significance level, or due to evidence that SAVVY effectively prevents HIV transmission. The Lan-Demets [16] spending function with O’Brien-Fleming type boundaries [17] will be employed to preserve the overall one-sided type I error rate for effectiveness at the 0.025 level, regardless of the timing of the interim analysis (the effective significance level for the interim analysis will be approximately 0.0026 if the interim analysis takes place at approximately the half-way point). Note that the two one-sided tests at the second interim analysis can also be thought of as a single two-sided test with asymmetric tail probabilities. Furthermore, the liberal significance level used to test for potential harm serves a dual purpose in that it also provides the DMC with a conservative guideline to allow consideration for terminating the study due to futility.

# 10.0 MONITORING PLAN SUMMARIES

## 10.1 Clinical Monitoring Plan

Site visits by a Clinical Monitor will be made in accordance with FHI standard operating procedures to monitor study operations, the quality of data collected in the research records, the accuracy of the data entered in the database, and to determine that all process and regulatory requirements are met. The monitoring will include assuring that the study is being conducted and informed consent is being obtained according to the protocol approved by the IRBs. The Monitor will make site visits on an approximate quarterly basis during the conduct of the study. A final closeout visit will be made approximately 6 weeks after the last participant has finished the study. A complete monitoring plan will be developed before study initiation.

Investigators will allow the monitors to inspect study documents (e.g., consent forms, drug distribution forms, data collection forms) and pertinent hospital or clinic records for confirmation of the study data.

## 10.2 Data Monitoring Committee

FHI will assemble an independent DMC made up of at least three members; we plan to include at least one clinician and one statistician. The DMC will meet once before the study begins and twice during the study (see Protocol Addendum, p.36). The details for the operation and responsibilities of the DMC will be provided in the DMC Operational Plan.

# 11.0 DATA MANAGEMENT PLAN SUMMARY

A detailed data management plan will be written prior to study initiation. It will be modified if significant changes are required, in order to document how data were handled in the study. The following is a brief summary of the plan.

Study sites will capture clinical data on 2-ply paper data collection forms (DCFs). The Investigator is responsible for the accuracy of data entered on the DCF. The original will be kept in the participant’s original file folder and the other copy will be used as a working copy for data entry and filed in the participant’s working file folder when entry is complete.

The sites will connect to an FHI secure server via the Internet using Citrix software and log directly into FHI's 21 CFR Part 11-compliant data management system. Citrix software allows a user to connect to a host computer and perform processing on the host computer, thus eliminating local software configuration concerns. Once the site data entry staff have logged onto FHI's secure network, they will use the ENTER module of the Clintrial™ data base management system (vendor software provided by Phase Forward, Inc.) The site data entry staff will double enter data (i.e., entry is performed by two independent system users) directly into the Clintrial™ data base. Subsequent data management processing (e.g., query generation, medical coding, data set creation) will take place in the Part 11-compliant environment at FHI.

If data entered on the DCFs are taken from an external source (e.g., laboratory reports, patient records), the source documents should be maintained in the participant’s medical chart or study file at the site, and should be available for review.

Data queries will be generated at FHI and sent to the Investigator every two weeks. The Investigator should answer queries within one week of receipt. The data relevant to the query will be updated based on the Investigator’s written response. Investigators must keep copies of all queries stapled to the DCFs in the participant’s file.

# 12.0 PROTOCOL VIOLATIONS

Emergency departures from protocol that eliminate an apparent immediate hazard to participants and are deemed crucial for the safety and well being of that participant may be instituted for that participant only – the Investigator will contact FHI as soon as possible. The Investigator will notify the IRB and FHI Project Leader in writing as soon as possible and document on the DCF reasons for deviation and ensuing events. Some examples of protocol violations include:

- Omission or inadequate administration of informed consent
- Inclusion/exclusion errors, including legal age limit
- Treatment errors: no treatment or incorrect treatment, including dose or regimen, or expired product
- Missing or incorrectly timed study procedures and assessments
- Forcing a participant to enter or remain in the study
- Participants who should have been discontinued from the study due to protocol criteria, but were not.

# 13.0 STUDY DOCUMENTS

## 13.1 Study Initiation

The following documents will be in place and monitored by a Clinical Monitor at the study site before any potential participants are contacted.

- Investigator’s Brochure
- FDA Form 1572
- Signed protocol and amendments
- Sample DCFs
- Financial disclosure forms for Investigator and Sub-Investigator
- Information given to participants
  - Consent forms
  - Other written information
  - Advertisements
- Financial records
- Signed agreements between involved parties
- IRB approval
- IRB composition
- Regulatory authority approval
- CV for Investigator, Sub-Investigator, and Site Coordinator
- Laboratory test normal values
- Lab accreditation and lab supervisor’s CV
- Instructions for handling of investigational product and trial related materials
- Shipping records
- Decoding procedures for masked trials
- Site Visit Log

## 13.2 Study Conduct

During the study the following documents will be in place and periodically monitored by a Clinical Monitor at the study site. Revision of documents will be made if relevant.

- Revisions to the Investigator’s Brochure
- Revisions to the FDA form 1572
- Revision to the Financial Disclosure forms
- Revision to the protocol and amendments
- Revisions to the DCFs
- Revisions of information given to participants
  - Consent forms
  - Other written information
  - Advertisements
- IRB approvals
- IRB composition changes
- Regulatory authority approvals
- CV for Investigator, Sub-Investigator, and Site Coordinator
- Updates of laboratory test normal values
- Documentation of investigational product and trial related materials shipment
- Relevant communications other than site visits
- Signed consent forms
- Source documents
- Copies of completed DCFs
- Copies of documentation of DCF corrections
- Notification of Investigator to FHI of SAEs
- Notification of Investigator to local IRB and Regulatory Authorities
- Notification by FHI or Sponsor to Investigator of safety information
- Interim or annual reports to IRB, Regulatory Authorities, and FHI
- Participant screening log
- Participant ID code list
- Participant enrollment log
- Investigational products accountability documents
- Staff signature list
- Site Visit Log
- Progress Reports to FHI

## 13.3 Study Completion

After completion of the study, all of the documents in 13.1 and 13.2 should be in the file together with the following:

- Investigational products accountability documents
- Documentation of products destruction (if destroyed at site)
- Complete participant ID code list
- Final report by Investigator to IRB, Regulatory Authorities, and FHI

## 13.4 Site Record Retention and Access to Documents at the Site

The signed original informed consent documents for each participant, yellow copies of DCFs, and originals of all study documentation (e.g. drug inventory forms, participant clinic records, original laboratory reports, diaries, etc.) will be retained by the Investigator for a minimum of two years after USFDA approval or withdrawal of a New Drug Application (NDA). If no NDA is submitted within five years of the last follow-up visit, the center may request permission in writing from FHI to destroy the records. **No records may be destroyed without written permission from FHI.**

The investigator may be subject to a field audit by FHI, Biosyn, or FDA inspectors to validate the participation of study participants and to verify the data reported on the DCFs. This audit could occur while the study is in progress, several years after the study is completed, or when the data are under review by the FDA as part of the pre-market approval process. All of the participants’ records and other study documentation must be filed and accessible on short notice (3-5 days) during the study and subsequent retention period.

# 14.0 QUALITY ASSURANCE

A Quality Assurance Associate from FHI will conduct a site visit and evaluation after at least 5 months of study conduct and then again about 6 months before the projected end of the study. The QA Associate will evaluate study systems for quality control, monitoring of the study, documentation of the study, and overall control of the study.

# 15.0 ETHICS AND RESEARCH INTEGRITY

## 15.1 Institutional Review Board Review and Approval

Before initiating the study, the study must be approved in writing by the Institutional Review Board at the site and FHI’s Protection of Human Subjects Committee in accordance with USFDA regulations (21 CFR Part 56). The study will be conducted in accordance with all conditions of approval by the IRBs. The sites will obtain written reapproval of the research by the IRBs at least annually and forward a copy of the review to FHI.

## 15.2 Informed Consent

No participant may be admitted into this study until the Investigator has obtained her legally effective informed consent. An Investigator shall seek such consent only under circumstances that provide the prospective participant with sufficient opportunity to consider whether or not to participate in the study. Informed consent must be obtained without coercion, undue influence, or misrepresentation of the potential benefits or risks that might be associated with participation in the study.

Informed consent encompasses all oral or written information given to the participant about the study and the study materials. This includes the consent form signed by the participant, recruitment advertising, and any other information provided to the participant. All such information that is given to the participant will be in a language that is understandable to her. The information will not include any language in which the participant is made to waive any of her rights or which releases or appears to release the Investigator, the Investigator’s institution, or FHI from liability for negligence.

Informed consent will be documented by the use of a written consent form that is signed by the participant (or the participant’s mark if she cannot sign). A copy of the signed consent form will be given to each participant. The original signed consent form for each participant will be kept in the participant’s study files separate from the DCFs. The consent form must include each of the basic and additional elements of informed consent described in 21 CFR Part 50.25 and must describe each of the risks or discomforts to the participant that have been identified by FHI as reasonably foreseeable.

## 15.3 Participant Confidentiality

The confidentiality of all participants enrolled into this study will be protected to the fullest extent possible. FHI staff, Biosyn staff, FDA personnel, or other individuals authorized in writing by FHI may audit participant’s clinic records. However, study participants should not be identified by name on any DCF or on any other documentation sent to FHI and will not be reported by name in any report or publication resulting from data collected in this study.

## 15.4 Research Integrity

Concern in the United States about the quality of biomedical and behavioral research has led to the establishment of regulations and guidelines for handling the allegations of scientific misconduct.  As a recipient of U.S. government funding, FHI is required to develop policies and procedures related to scientific misconduct that conform to these regulations.  The regulations define "misconduct" in science as a "fabrication, falsification, plagiarism, or other practices that seriously deviate from those that are commonly accepted within the scientific community for proposing, conducting, or reporting research.  It does not include honest errors or honest differences in interpretations or judgments of data (42 CFR 50.102.)

FHI has specific obligations under these regulations with respect to the handling of information related to scientific misconduct that may come to its attention.  In addition, the regulations require that FHI establish procedures related to initiating an inquiry, pursuing an investigation, and informing a specified circle of authorities should the situation warrant it.  FHI will pursue all allegations of misconduct in research or questionable academic conduct that may raise legitimate suspicions of misconduct, and will conduct inquiries and investigations to resolve questions regarding the integrity of research.  In conducting inquiries during an investigation, FHI will focus on the substance of the issue and will be vigilant not to permit personal conflicts between colleagues to obscure the facts.

# 16.0 PUBLICATION POLICY

All information concerning the drug supplied by FHI to the Investigator and not previously published is considered confidential.

The initial publication from this study will be the aggregate multicenter results focusing on the primary endpoints of SAVVY safety and HIV prevention effectiveness. Subsequent secondary analyses and manuscripts, including any single center data, will be agreed upon by members of the protocol team and reviewed by FHI. All suggestions for the manuscript will be returned to the first author by FHI within 15 working days of receiving it.

For publication of the main findings of this study, the Investigator and Study Coordinator from each site, and the Project Leader and Lead Statistician from FHI will be considered as authors (a total of 6 persons). Other key contributors to the research will be acknowledged as part of the Nigeria SAVVY Phase 3 Research Team.

# 17.0 ADDITIONAL INVESTIGATOR RESPONSIBILITIES

In addition to the previously described responsibilities, the Investigator is responsible for signing and dating the cover page of this study protocol. The signed and dated original must be submitted to FHI before protocol implementation, and the Investigator at the site must maintain a copy with the study files.

All protocol amendments must be signed and dated by the Investigator. The signed and dated original must be submitted to FHI before implementation of the amendment, and the Investigator at the site must maintain a copy. Amendments must be approved by FHI and the IRB before implementation.

The Investigator will provide FHI with a curriculum vitae (CV) for himself showing the education, training, and experience that qualifies him as an expert in the area of clinical investigation specific to the product under investigation and his affiliation with the site at which the study is being conducted. CVs also must be provided for all individuals listed as Sub-Investigators on the Form FDA-1572 and the Site Coordinator showing the education, training, and experience that qualifies them for their role in the study, and their affiliation with the study site. If and when any personnel listed on the 1572 changes, the Investigator will notify FHI and provide a CV for any new staff on the form.

The Investigator will supply FHI with copies of the current license and/or laboratory certification (such as the Clinical Laboratory Improvement Act [CLIA] certification) of any laboratory used for the study. The Investigator is responsible for obtaining any updates to these documents and sending them to FHI in a timely fashion. This includes documentation of the normal ranges of the laboratory tests used by the laboratory. The Investigator will ensure that appropriate health care or referral is provided for the study participants throughout the study. Copies of all these documents must be maintained at the site.

Throughout the course of the study, the Investigator will prepare and submit to FHI whatever reports are required and detailed in the Subcontract Agreement to be signed prior to initiating the study. These reports summarize the accomplishments of the assignment and usually include the following:

- all changes in the research activity; and
- all unanticipated problems involving risks to participants or others.

The final technical report will be submitted within 45 days after completion of the study unless this period is extended in writing by FHI.

# 18.0 REFERENCES

1. Joint United Nations Programme on HIV/AIDS. Report on the Global HIV/AIDS Epidemic 2002.
2. Wortley PM, Fleming PL. AIDS in women in the United States. Recent trends. JAMA 1997;278:911-916.
3. Haynes BF. HIV vaccines: where we are and where we are going. *Lancet* 1996;348:933-937.
4. Centers for Disease Control. Update: barrier protection against HIV infection and other sexually transmitted diseases. *MMWR* 1993;42**:** 589-597.
5. Stone AB, Hitchcock PJ. Vaginal microbicides for preventing the sexual transmission of HIV. *AIDS* 1994;8**:**S285-S293.
6. Elias CJ, Coggins C. Female-controlled methods to prevent sexual transmission of HIV. *AIDS* 1996;10:S43-51.
7. Elias CJ, Heise LL. Challenges for the development of female-controlled vaginal microbicides [editorial]. *AIDS* 1994;8:1-9.
8. The International Working Group for Vaginal Microbicides. Recommendations for the development of vaginal microbicides. *AIDS* 1996;10:UNAIDS1-UNAIDS6.
9. Niruthisard S, Roddy RE, Chutivongse S. The effects of frequent nonoxynol-9 use on the vaginal and cervical mucosa. *Sex. Transm. Dis.* 1991;18:176-179.
10. Roddy RE, Cordero M, Cordero C, Fortney JA. A dosing study of nonoxynol-9 and genital irritation. *Int. J. STD AIDS* 1993;4:165-70.
11. Biosyn, Inc. Investigator’s Brochure. October 2002.
12. Corner AM, Dolan MM, Yankell SL, and Malamud D.C31G, a new agent for oral use with potent antimicrobial and antiadherence properties. *Antimicrobial Agents and Chemotherapy* 1988;32:350-353.
13. Calis S, Yulug N, Sumnu M, Ayhan A, and Hincal AA. A non-antibiotic antimicrobial mixture (C31G): evaluation of the antimicrobial efficiency of C31G on vaginal cultures" *Boll. Chim. Farmaceutico* 1992;131:335-338.
14. Thompson KA, Malamud D, and Storey BT. Assessment of the anti-microbial agent C31G as a spermicide: comparison with nonoxynol-9. *Contraception* 1996;53:313-318.
15. Stieritz DD, Bondi A, McDermott D, Michaels ED. A burned mouse model to evaluate anti-Pseudomonas activity of topical agents. *J Antimicrob Chemother* 1982;2:133-40.
16. Sondo B, Testa J, Kone B. The use of contraceptive methods by the Mossi in a rural health district of Kaya, Burkina Faso. *Sante* 2001;2:111-6.
17. Lan, KKG, and DeMets, DL. Discrete Sequential Boundaries for Clinical Trials. Biometrika, 1983;70:659-663.
18. O’Brien, PC and Fleming, TR. A multiple testing procedure for clinical trials. *Biometrics,* 1979;35:549-56.

# PROTOCOL ADDENDUM

**Study 9784: Randomized Controlled Trial of SAVVY and HIV in Nigeria**

Amendment 2

After careful discussion with the manufacturer of SAVVY gel, other microbicide researchers, and women’s health advocates, we will increase the level of safety monitoring for participants in the SAVVY phase 3 trials. Our intensified safety monitoring will involve four activities that obligate a study protocol amendment.

**1. Statistical Monitoring Plan and DMC Review**

The following represents our proposed statistical plan for monitoring the SAVVY trials for a potentially harmful SAVVY effect. However, the final plan is contingent upon review and approval by the sponsor and the members of the DMC.

In addition to the interim analysis for both harm and effectiveness at the study midway point (i.e., after approximately 33 HIV events) as originally planned, we propose to perform a first interim analysis after approximately 16 HIV events have occurred. After 16 events, we will ask that the DMC only consider stopping the study for a potentially harmful SAVVY effect (not because of early evidence that SAVVY is effective). At the study midway point, the DMC will again look for a potentially harmful effect, but we will also allow consideration for stopping the study due to significant evidence that SAVVY is effective at that interim point. The test that we propose to use at the midway point will be asymmetric, making it easier to stop for potential harm than for effectiveness.

For each of the two interim analyses, we propose to use a more liberal significance level (e.g., 0.10) to test for a potentially harmful effect. Statistical simulations and power calculations show that conducting a one-sided test for harm at the 0.10 significance level at each of the two interim analyses would have the following properties/operating characteristics:

- would scarcely reduce the power to detect a significant positive effect of SAVVY
- would not inflate the probability of a type I error for effectiveness
- would not increase the necessary study size
- would be able to detect a harmful effect of SAVVY (relative rate = 2) with 55% power at the first interim look (after only 16 HIV events) and with 80% power by the halfway point.

It is important to recognize that the proposed test for a potentially harmful effect will not be likely to offer conclusive evidence that SAVVY is indeed harmful relative to placebo. For example, if SAVVY truly has no effect beyond that of the placebo, then the proposed test at the 0.10 significance level has a 10% probability of concluding "potential harm" at the first look and a 15% chance of concluding “potential harm” at either the first or second look. Thus, before recommending that either study should be terminated early due to interim evidence of a potentially harmful effect, the DMC will likely want to consider the emerging evidence from the other study as well. So, for example, if the interim analysis in the Nigeria study after 16 events resulted in a marginally significant p-value of 0.09 but the interim results from the Ghana study indicated that SAVVY may well be protective, then it may be unwise for the DMC to recommend stopping either study. In addition, the DMC will likely consider other evidence from within each of the studies (such as the distribution of AEs across the treatment arms) before making their recommendation.

In order to maintain the overall one-sided significance level for declaring SAVVY effective at 0.025, we will adjust the effectiveness significance level at the interim and final looks using the Lan-DeMets spending function with O'Brien-Fleming type boundaries, as specified in the protocol. If the interim analysis occurs at precisely the halfway point in terms of number of HIV infections, the one-sided significance levels to test for effectiveness will be 0.0026 and 0.024 at the interim and final analyses, respectively.

**2. Additional Scheduled Testing for Chlamydial Infection**

The USFDA Division of Antiviral Drug Products has expressed concern that we could miss a large number of cervical infections, given that study participants would make their follow-up visits at outreach offices and no regular testing would be scheduled after baseline. That way, only symptomatic (or simply worried) women would be directed to the clinics for STI testing. The Sponsor is not aware of any reliable data to suggest that use of SAVVY increases the risk of chlamydia, and additional clinic visits with pelvic examinations could increase participant burden/fatigue, which in turn might compromise our retention rates and ability to assess effectiveness against HIV acquisition.

To address the USFDA concern without threatening retention unduly, we propose to test each study participant one more time during the study, at or after her six-month observation point. The chlamydia testing requires a cervical swab and thus a clinic visit. With the large number of women in this study, we will have 80% power to detect a roughly 5% difference in prevalence using a logistic regression model that adjusts for baseline STI status.

The Sponsor is not aware of any reliable data indicating reduced test performance in the presence of SAVVY gel. In addition, the characteristics of the gel substance would not be expected to interfere or cross react with the antigen capture technology employed in the study chlamydia test. However, the sponsor has decided to perform further validation studies of chlamydia test performance in the presence of study gel to yield additional data on the accuracy of such testing.

The follow-up chlamydia data, and the information on chlamydia test performance, will allow us to evaluate whether women in one of the study arms have a higher prevalence of chlamydial infection, a possible indicator of harm. These data will be made available to the DMC, along with the HIV incidence data and information on other adverse events (AEs).

1. **Ascertainment of Pregnancy Outcomes**

We know that a number of pregnancies will occur during the SAVVY studies. We will counsel women who become pregnant to stop using the gel, although they will be encouraged to continue making follow-up visits. We have not included items on pregnancy outcomes on our data collection forms. The FDA has asked Biosyn to supply data on outcomes of pregnancies occurring while women are using study product.

In response, we will design a worksheet to capture information on the pregnancy outcomes. The worksheet will not be a data collection form entered into the data base, but rather a simple single sheet for each pregnancy. This task will be done by the outreach workers. In some cases, outcome data will be collected at routine follow-up visits. In other cases, outreach workers will have to make home visits for a pregnancy outcome interview. We will summarize these outcomes for all participants who become pregnant during the study.

We will develop a financial arrangement that allows some outreach workers to be retained on staff long enough to follow all of the pregnancies that occur during the study, even those that end after the trial is finished.

1. **Adverse Event Monitoring**

In addition to the above protocol changes, we will monitor quarterly or more frequently all adverse events (AEs) entered in the Ghana and Nigeria study data bases by our web-based data entry procedures. AEs will be coded using the MEDRA system in a collaborative effort by the study clinician and data management staff. AEs will then be summarized by body system, but not by study arm. Incidence and prevalence rates will be calculated, but no statistical comparisons will be made.

These routine AE monitoring procedures are in addition to standard Serious Adverse Event (SAE) reporting that will be performed as required by the protocol, the USFDA, and the IRBs.

Finally, the SAVVY trials will follow revised FHI SOP #301-04 on SAE handling. The main change in this SOP is that SAEs will be directed to Regulatory Affairs and Quality Assurance (RAQA) staff first, in an effort to speed the assessment and reporting of SAEs to the sponsor and the USFDA. Study staff will be trained to use the updated SAE fax cover sheet and to direct the SAE reports to the RAQA fax machine.

# APPENDIX 1: STUDY ACTIVITIES CHART

| Event | **Screening** | **Enrollment** | **Follow-Up** | **6-month** | **Final Visit** |
| --- | --- | --- | --- | --- | --- |
| **Informed consent** | ◆ | ◆ | ◆ |  |  |
| **Eligibility criteria assessment** | ◆ | ◆ | ◆ |  | ◆ |
| **Assign Participant ID number** | ◆ |  |  |  |  |
| **Pre-test HIV counseling** | ◆ | ◆ | ◆ | ◆ | ◆ |
| **HIV test*** | ◆ | ◆ | ◆ | ◆ | ◆ |
| **Syphilis serology** | ◆ | (if indicated) | (if indicated) | (if indicated) | (if indicated) |
| **Urine pregnancy test** | ◆ | ◆ | ◆ | ◆ | ◆ |
| **Obtain contact information** | ◆ | ◆ | ◆ |  |  |
| **Pelvic exam** | ◆ | (if indicated) | (if indicated | ◆ | (if indicated) |
| **Vaginal wet mount** | ◆ | (if indicated) | (if indicated) | ◆ | (if indicated) |
| **Gonorrhea** | ◆ | (if indicated) | (if indicated) | (if indicated) | (if indicated) |
| **Chlamydia Test** | ◆ | (if indicated) | (if indicated) | ◆ | (if indicated) |
| **Counseling for STI prevention** | ◆ | ◆ | ◆ |  | ◆ |
| **Interview**** | ◆ | ◆ | ◆ |  | ◆ |
| **Post-test HIV counseling & test results** | ◆ | ◆ | ◆ | ◆ | ◆ |
| **AE assessment** |  |  | ◆ | ◆ | ◆ |
| **Concomitant medications** | ◆ | ◆ | ◆ | ◆ | ◆ |

* A blood specimen will be collected during the enrollment visit and stored for later confirmatory HIV testing if that participant seroconverts during the follow-up period. Additionally, a blood specimen will be collected at the final visit for further HIV testing.

**Interview includes an acceptability questionnaire at the final visit.

# APPENDIX 2: DIRECTIONS FOR USE OF STUDY GEL

Instructions on Gel Use

**Please read the following directions carefully before use:**

1) Wash your hands with soap and water before you use either of the study products.

2) Insert plunger into prefilled applicator by placing small end of plunger into the open hole at the end of the applicator and push until you feel it snap into place. Do not push it past that point.

3) Pull off the blue cap from the prefilled applicator.

4) Hold the prefilled applicator by the opposite end from the where the gel is. Gently insert the

applicator into the vagina as far as it will go comfortably. Push the plunger in to expel the gel.


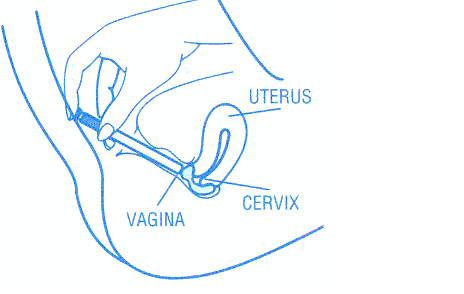


Insertion of the applicator is accomplished more easily when lying on your back with the knees bent.

5) Throw away the applicator after each use. Do not flush in toilet.

# APPENDIX 3: HANDLING AND STORAGE INSTRUCTIONS

The product should be stored at Controlled Room Temperature, which as defined by USP, encompasses the usual and customary temperature range of 20º - 25ºC (68º - 77ºF), and allows for excursions between 15º and 30ºC (59º - 86ºF), and transient spikes up to 40ºC (104ºF) for less than 24 hours. The mean kinetic temperature (time average) over the duration of storage should not exceed 25ºC (77ºF).

Shipping cartons and patient packages have been designed to be compatible with normal shipping and handling procedures. It should be visually noted that no packages have unexpectedly received damage during shipment or handling.

# APPENDIX 4: PARTICIPANT FLOW

# Participant Flow Through the Study

Potential participant contact with outreach staff

Eligible women referred to study clinic

Screening study:

consent, and HIV testing

Participants return in one month for enrollment

Enrollment: consent, HIV testing, and randomization

12 Monthly Follow-up Visits

Follow-up: consent and HIV testing

1. The protocol originally described this same testing as two-sided. However, since the type I error rate will be tightly controlled at 0.025 for detecting a protective effect of the SAVVY gel, but will be greater than 0.025 for detecting a harmful effect of SAVVY, the testing is now being described as two one-sided tests. [↑](#footnote-ref-2)
